# *Francisella tularensis* Susceptibility to Antibiotics: A Comprehensive Review of the Data Obtained *In vitro* and in Animal Models

**DOI:** 10.3389/fcimb.2017.00122

**Published:** 2017-04-11

**Authors:** Yvan Caspar, Max Maurin

**Affiliations:** ^1^Laboratoire de Bactériologie-Hygiène Hospitalière, Département des agents infectieux, Centre National de Référence des Francisella, Institut de Biologie et de Pathologie, Centre Hospitalier Universitaire Grenoble AlpesGrenoble, France; ^2^Université Grenoble Alpes, Centre National de la Recherche Scientifique, TIMC-IMAGGrenoble, France

**Keywords:** *Francisella tularensis*, antimicrobial susceptibility, MIC, MBC, intracellular, animal model, tularaemia, antibiotic therapy

## Abstract

The antibiotic classes that are recommended for tularaemia treatment are the aminoglycosides, the fluoroquinolones and the tetracyclines. However, cure rates vary between 60 and 100% depending on the antibiotic used, the time to appropriate antibiotic therapy setup and its duration, and the presence of complications, such as lymph node suppuration. Thus, antibiotic susceptibility testing (AST) of *F. tularensis* strains remains of primary importance for detection of the emergence of antibiotic resistances to first-line drugs, and to test new therapeutic alternatives. However, the AST methods reported in the literature were poorly standardized between studies and AST data have not been previously evaluated in a comprehensive and comparative way. The aim of the present review was to summarize experimental data on antibiotic susceptibilities of *F. tularensis* obtained in acellular media, cell models and animal models since the introduction of fluoroquinolones in the treatment of tularaemia in 1989. We compiled MIC data of 33 antibiotics (including aminoglycosides, fluoroquinolones, tetracyclines, macrolides, β-lactams, chloramphenicol, rifampicin, and linezolid) against 900 *F. tularensis* strains (504 human strains), including 107 subsp. *tularensis* (type A), 789 subsp. *holarctica* (type B) and four subsp. *mediasiatica* strains, using various AST methods. Specific culture media were identified or confirmed as unsuitable for AST of *F. tularensis*. Overall, MICs were the lowest for ciprofloxacin (≤ 0.002–0.125 mg/L) and levofloxacin, and ranged from ≤ 0.016 to 2 mg/L for gentamicin, and 0.064 to 4 mg/L for doxycycline. No resistant strain to any of these antibiotics was reported. Fluoroquinolones also exhibited a bactericidal activity against intracellular *F. tularensis* and lower relapse rates in animal models when compared with the bacteriostatic compound doxycycline. As expected, lower MIC values were found for macrolides against type A and biovar I type B strains, compared to biovar II type B strains. The macrolides were more effective against *F. tularensis* grown in phagocytic cells than in acellular media.

## Search strategy and selection criteria

Data on antibiotic susceptibility testing (AST) of *Francisella tularensis*, both *in vitro* and in animal models, were collected from the English and French literature in the PubMed database, since the introduction of fluoroquinolones in the treatment of tularaemia in 1989 until December 2016. They were extracted using the key words “tularemia” and “*Francisella*” in order to achieve a broad screening of the entire body of literature on the subject since 1989. Only studies evaluating more than five *F. tularensis* strains were selected for analysis.

## *Francisella tularensis* and tularemia

*F. tularensis*, the etiological agent of tularaemia, is a Tier 1 biological threat agent according to the classification of the Centers for Disease Control and Prevention (Dennis et al., 2001). It was first isolated from ground squirrels in 1911 in Tulare County, California (USA), and from a human tularaemia case in 1914 in Ohio (USA) (Mccoy and Chapin, 1912; Wherry and Lamb, 1914). The name “*Francisella tularensis*” was coined in 1959 to honor Dr. Edward Francis, who greatly contributed to improve the knowledge on human tularaemia (Francis et al., 1922; Francis, 1928; Rockwood, 1983). *Francisella tularensis* is currently divided into three subspecies: subsp. *tularensis* (type A strains), mainly found in North America; subsp. *holarctica* (type B strains) found throughout the northern hemisphere; and subsp. *mediasiatica* found in Central Asia (Olsufiev et al., 1959; Jellison and Owen, 1961; Olsufjev, 1970; Jellison, 1974; Olsufjev and Meshcheryakova, 1983). Debate continues on whether *Francisella novicida* is a fourth subspecies of *F. tularensis* or a separate species, but we agree with Johansson et al. in keeping *F. novicida* in a separate species because of its aquatic reservoir and very low virulence in humans (Busse et al., 2010; Huber et al., 2010; Johansson et al., 2010). Type B strains are also classically differentiated into three biovars (Kudelina and Olsufiev, 1980; Olsufjev and Meshcheryakova, 1982, 1983): biovar I (naturally susceptible to erythromycin) is found in Western Europe and North America; biovar II (naturally resistant to erythromycin) is found in Eastern Europe and Asia; and biovar japonica (susceptible to erythromycin but fermenting glycerol) is mainly found in Japan, although it has recently been described in China and Turkey (Kiliç et al., 2013; Wang et al., 2014). Biovar II strains are found in the Eastern part of Europe (Czech Republic, Finland, Georgia, Russia, Slovakia, Ukraine, Austria, Hungary, and Romania) (Vogler et al., 2009; Svensson et al., 2009b; Chanturia et al., 2011; Gyuranecz et al., 2012) and in Asia and both biovar I and II strains coexist in Germany, Switzerland and Scandinavia (Muller et al., 2013; Origgi et al., 2014; Maurin and Gyuranecz, 2016).

Although the *F. tularensis* genome displays very low variability, four distinct clades have been identified by pulse-field gel electrophoresis (PFGE) within type A strains in the United States (A.Ia, A.Ib, A.IIa, and A.IIb) with the A.Ib clade being associated with a 24% mortality rate in humans (Kugeler et al., 2009). Type B strains have also been divided into several clades by whole genome sequencing. The four main clades include clade B.4 corresponding to North American strains, clade B.6 to biovar I Western European strains, clade B.12 to biovar II Eastern European strains, and clade B16 to strains belonging to biovar japonica (Fujita et al., 2008; Vogler et al., 2009; Kilic et al., 2015; Karlsson et al., 2016).

*F. tularensis* is a Gram-negative, facultative intracellular coccobacillus (Broman et al., 2011). It is strictly aerobic, non-motile, non-toxigenic, and non-spore-forming. It is a fastidious bacterium that may be grown in cysteine-enriched media, under 5% CO_2_ atmosphere. The main virulence factor of *F. tularensis* corresponds to its ability to multiply within eukaryotic cells, especially in the cytosol of macrophages (Clemens and Horwitz, 2007). Virulence is associated with the presence of a duplicated pathogenicity island in the bacterial genome, encoding a type VI-like secretion system (Nano and Schmerk, 2007). However, the high variations in virulence observed among *F. tularensis* genotypes remain currently unexplained.

Human tularaemia is a zoonotic disease usually occurring as sporadic cases or small familial outbreaks (Tärnvik and Berglund, 2003; Bicakci and Parlak, 2008). However, a number of tularaemia outbreaks have been reported, including in the last two decades (Helvaci et al., 2000; Cerný, 2001; Feldman et al., 2001; Pérez-Castrillón et al., 2001; Reintjes et al., 2002; Christova et al., 2004; Payne et al., 2005; Celebi et al., 2006; Kantardjiev et al., 2006; Siret et al., 2006; Petersen et al., 2008; Akalın et al., 2009; Barut and Cetin, 2009; Svensson et al., 2009a; Hauri et al., 2010; Mailles et al., 2010; Larssen et al., 2011, 2014; Wang et al., 2011; Karlsson et al., 2013; Johansson et al., 2014; Mengeloglu et al., 2014). Humans may be infected with *F. tularensis* through direct contact with infected animals (manipulation of live or dead infected animals, animal bites or scratches), consumption of contaminated food or water, exposure to contaminated environments or through arthropod bites (Keim et al., 2007). *F. tularensis* can infect a large number of animal species, but lagomorphs and small rodents are considered key hosts for this pathogen (Gyuranecz et al., 2011; Maurin and Gyuranecz, 2016). This bacterium may also persist in water and soil environments for several months, which might be related to an ability to multiply in protozoa, such as amoebae (Abd et al., 2003; Keim et al., 2007; El-Etr et al., 2009; Broman et al., 2011). A terrestrial and an aquatic lifecycle have been described for this bacterium (Maurin and Gyuranecz, 2016). Arthropods, such as ticks and mosquitoes may be contaminated by *F. tularensis* from the animal or environmental reservoirs (Sjostedt, 2007; Maurin and Gyuranecz, 2016). Large tularaemia outbreaks have occurred, for which multiple sources of contamination and several *F. tularensis* clones were identified (Akalın et al., 2009; Barut and Cetin, 2009; Celebi et al., 2006; Cerný, 2001; Christova et al., 2004; Feldman et al., 2001; Hauri et al., 2010; Helvaci et al., 2000; Johansson et al., 2014; Kantardjiev et al., 2006; Karlsson et al., 2013; Larssen et al., 2011, 2014; Mailles et al., 2010; Mengeloglu et al., 2014; Payne et al., 2005; Pérez-Castrillón et al., 2001; Petersen et al., 2008; Reintjes et al., 2002; Siret et al., 2006; Svensson et al., 2009a; Wang et al., 2011). Symptoms vary according to the infection route and classically correspond to six different clinical forms: ulceroglandular, glandular, oropharyngeal, oculoglandular, pneumonic and typhoidal (Tarnvik, 2007). Ulceroglandular and glandular forms are consecutive to skin inoculation of bacteria (e.g., through an arthropod bite or contact with infected animals). A regional lymphadenopathy develops with (ulceroglandular form) or without (glandular form) a skin ulcer at the inoculation site. When contamination occurs through the ocular conjunctiva, painful conjunctivitis with regional lymphadenopathy develops, which corresponds to oculoglandular tularaemia. The oropharyngeal form is characterized by pharyngitis with regional lymphadenopathy, usually occurring after consumption of contaminated meat or water. Pneumonic tularaemia results from airborne contamination or hematogenous spread of bacteria to the lungs and is characterized by unspecific symptoms, such as cough, fever, dyspnea and occasionally a mediastinal or hilar lymphadenopathy. The typhoidal form corresponds to systemic disease with neurological symptoms mimicking typhoid, often with *F. tularensis* bacteraemia, but without detection of any portal of entry of bacteria (especially no skin ulcer) and without symptoms of localized infection (especially no regional lymphadenopathy) (Tärnvik and Chu, 2007). Complications may occur, such as skin eruptions, abscess formation, lymph node suppuration with occasionally skin fistulisation, and secondary infectious locations due to hematogenous spread of bacteria (Tärnvik and Chu, 2007; Meric et al., 2008; Maurin et al., 2011; Erdem et al., 2014). Diagnosis is usually based on compatible clinical and epidemiological features, and positive serological tests. PCR is useful to detect *F. tularensis* DNA in various clinical samples, especially before specific antibodies can be detected (Tarnvik, 2007). Isolation of a *F. tularensis* strain from blood or tissue samples is obtained in <20% of patients, which makes antibiotic susceptibility testing (AST) of *F. tularensis* strains rare (Maurin et al., 2011).

The 2007 WHO guidelines for treatment of tularaemia are based on the 2001 consensus recommendations from Dennis et al. (2001). According to these publications, tularaemia treatment is mainly based on three antibiotic classes: the aminoglycosides, the fluoroquinolones and the tetracyclines, although many other antibiotic classes have been tested against *F. tularensis in vitro*. Here we have compiled all available experimental data on the evaluation of the activity of antibiotics against *F. tularensis in vitro*, in acellular and cell models, and *in vivo* in animal models to provide useful information for clinical microbiologists and physicians.

## Antibiotic susceptibility testing of *F. tularensis*

Antibiotic susceptibility testing (AST) of *F. tularensis* strains is usually not performed on a routine basis because: (1) isolation of the strain involved is only obtained in a few tularaemic patients (Maurin et al., 2011); (2) this procedure is at risk for the laboratory personnel handling cultures of this highly infectious pathogen and requires biosafety level 3 (BSL3) facilities (Tärnvik and Chu, 2007); (3) acquired resistance to antibiotics has never been reported so far in clinical strains of *F. tularensis* (Tarnvik, 2007) and (4) *in vitro* data may not be predictive of treatment failure in tularaemia patients. However, reference laboratories have reported AST surveys for collections of human and animal strains of *F. tularensis* (Scheel et al., 1993; Ikäheimo et al., 2000; Johansson et al., 2000, 2002; García del Blanco et al., 2004; Tomaso et al., 2005; Urich and Petersen, 2008; Valade et al., 2008; Velinov et al., 2011; Yeşilyurt et al., 2011; Georgi et al., 2012; Hotta et al., 2013; Kiliç et al., 2013; Kreizinger et al., 2013; Origgi et al., 2014).

For this review, we collected *F. tularensis* AST results from all studies of more than five tularaemia cases, published in the literature since 1989. We obtained data from 898 *F. tularensis* strains isolated from humans (510 strains), animals (200 strains), arthropods (four strains), natural water samples (39 strains) and unknown sources (147). They included 107 type A, 789 type B and four *F. tularensis* subsp. *mediasiatica* strains (Table 1 and Table S1) (Scheel et al., 1993; Ikäheimo et al., 2000; Johansson et al., 2000, 2002; García del Blanco et al., 2004; Tomaso et al., 2005; Urich and Petersen, 2008; Valade et al., 2008; Velinov et al., 2011; Yeşilyurt et al., 2011; Georgi et al., 2012; Hotta et al., 2013; Kiliç et al., 2013; Kreizinger et al., 2013; Origgi et al., 2014). AST results were available for 33 antibiotics, although only some of them were tested in all studies (Tables 1–**5** and Table S1). These antibiotics included four aminoglycoside compounds (gentamicin, streptomycin, amikacin and tobramycin), nine fluoroquinolones (ciprofloxacin, levofloxacin, moxifloxacin, ofloxacin, norfloxacin, grepafloxacin, sparfloxacin, trovafloxacin and gatifloxacin), three tetracyclines (tetracycline, doxycycline and tigecycline), four macrolides (erythromycin, azithromycin, roxithromycin and clarithromycin), one ketolide (telithromycin), nine β-lactams (ampicillin, amoxicillin + clavulanic acid, ceftazidime, piperacillin + tazobactam, ceftriaxone, cefepime, imipenem, meropenem and aztreonam), chloramphenicol, rifampicin, and linezolid.

**Table 1:** **Characteristics of *F. tularensis* strains included in this study and MICs for aminoglycosides (gentamicin and streptomycin), the tetracycline compound doxycycline and fluoroquinolones (ciprofloxacin and levofloxacin) according to the culture medium used and to the AST assay used**.

**Reference**	**Year(s)**	**Origin**	**Nb of strains Type/biovar**	**Culture medium**	**MIC (mg/L)**									
					**Gentamicin**		**Streptomycin**		**Doxycycline**		**Ciprofloxacin**		**Levofloxacin**	
					**Range**	**MIC_90_**	**Range**	**MIC_90_**	**Range**	**MIC_90_**	**Range**	**MIC_90_**	**Range**	**MIC_90_**
**BROTH MICRODILUTION**														
Origgi et al., 2014	1996–2013	Switzerland	19 B6	caMHB + 2% IsoVitaleX	≤ 0.12-0.25	0.25	4	4			≤ 0.06	≤ 0.06		
			5 B12		≤ 0.12–0.25		4				≤ 0.06			
Georgi et al., 2012	UNK	Europe	69 B	caMHB + 2% IsoVitaleX	≤ 0.25–0.5	0.5	≤ 0.5–2	2			≤ 0.031–0.125	0.063	≤ 0.031–0.125	0.063
		North America	7 A	caMHB + 2% IsoVitaleX	≤ 0.25–0.5		≤ 2				0.031–0.125			
		Central Asia	4 ssp. *mediasiatica*	caMHB + 2% IsoVitaleX	≤ 0.25		≤ 2				≤ 0.031–0.063			
Urich and Petersen, 2008	1974–2005	North America	92 A	caMHB + 2% IsoVitaleX	0.03–0.5	0.25	0.25–4	2	0.25–4	2	0.004–0.06	0.06	0.015–0.12	0.06
			77 B	caMHB + 2% IsoVitaleX	0.03–0.5	0.12	0.25–4	2	0.25–2	2	0.008–0.06	0.03	0.015–0.12	0.06
***E*****-TEST**														
Johansson et al„ 2000	1998	Sweden	7 B	Modified Thayer-Martin	0.5–1				0.25–0.5		0.008–0.015			
Hotta et al., 2013	1926–1989	Japan	36 B	Chocolate II agar	0.023–0.5	0.125			0.094–1.5	1	0.003–0.023	0.016		
Kreizinger et al., 2013	2003–2010	Hungary	29 B12	Modified Francis Agar	0.38–1	0.75	3–8	6	0.12–1.5	1	0.012–0.047	0.047	0.004–0.023	0.023
Yeşilyurt et al., 2011	2009–2010	Turkey	39 B biovar II	GBCA	0.094–0.25	0.25	0.75–1.5	1.5			0.008–0.016	0.016	0.006–0.016	0.012
Tomaso et al., 2005	1992–1998	Austrian	50 B biovar II	CHAB	0.094–2	0.75	0.75–8	3	0.38–3	2	0.004–0.125	0.032	0.008–0.047	0.032
Ikäheimo et al., 2000	UNK	Finland	38 B	CHA + 2% Haemoglobin	0.38–1.5	1	0.25–4.0	4			0.008–0.023	0.016	0.008–0.023	0.016
Kiliç et al., 2013	2009–2012	Turkey	249 B biovar II + 1 biovar *japonica*	CHAB	0.094–0.38	0.25	0.5–2	1.5	0.064–0.38	0.25	0.004–0.023	0.016	0.003–0.016	0.012
Johansson et al., 2002	1996–2001	USA	8 A	MHII + 1% IsoVitaleX or CHAB	0.032–0.25		0.064–2		0.125–2		0.016–0.064		0.016–0.064	
			16 B		0.016–0.125	0.064	0.064–1	0.25	0.125–2	1	0.016–0.064	0.064	0.008–0.125	0.125
Velinov et al., 2011	UNK	Bulgaria	21 B biovar II	caMHB + 2% IsoVitaleX	0.064–0.5	0.125	0.25–2	1	0.25–4	2	0.002–0.06	0.047	0.016–0.125	0.094
**AGAR DILUTION**														
Valade et al., 2008	1996–2005	France	71 B	MHII + 2% IsoVitaleX	0.03–0.5		<0.5–1		0.125–1		0.015–0.03			
Johansson et al., 2000	1998	Sweden	7B	Modified Thayer-Martin	1				0.5		0.03			

*caMHB, cation-adjusted Mueller Hinton Broth; GBCA, glucose blood cystein agar; MHII, Mueller Hinton II; CHA, cystein heart agar; CHAB, cystein heart agar enriched with blood; MIC_90_, minimal inhibitory concentration that inhibits 90% of the strains; MIC range, range between the lowest and the highest MIC observed*.

### MICs: methods and results

According to the Clinical and Laboratory Standards Institute (CLSI) and WHO guidelines, AST of *F. tularensis* strains should be performed using cation-adjusted Mueller-Hinton broth enriched with 2% defined growth supplement, such as Polyvitex® (referred to here as enriched caMHB), in order to fulfill the cystein growth requirement of this bacterium (Tarnvik, 2007; CLSI. M45-A2, 2010). Adjustment of pH to 7.1 ± 0.1 is mandatory after addition of 2% growth supplement because it significantly reduces the pH of caMHB medium. The bacterial inoculum must be calibrated at 5 10^5^ CFU/mL of final concentration. Culture media should be incubated for 48 h in ambient air, but incubation in 5% CO_2_-enriched atmosphere may be needed for some strains, although it can lead to acidification of the culture medium and therefore overestimation of aminoglycoside and macrolide MICs, or underestimation of tetracyclines MICs (CLSI. M45-A2, 2010).

In the past three decades, three different methods for *F. tularensis* AST were most frequently reported in the literature: antibiotic agar dilution, broth microdilution and E-test strips. These techniques were poorly standardized between studies, including after CLSI guidelines were available (CLSI. M45-A2, 2010). Most particularly, growth media, incubation conditions (atmosphere and duration) and bacterial inocula used for *F. tularensis* AST greatly varied between studies. Therefore, the results obtained in these different studies should be compared with caution.

As for the broth microdilution method, a number of authors used Mueller-Hinton II broth supplemented with glucose, Ca^2+^ and Mg^2+^ ions, and ferric pyrophosphate (a medium referred to as modified MHII) in spite of the recommended enriched caMHB medium (Baker et al., 1985; García del Blanco et al., 2004; Origgi et al., 2014). The use of MHII resulted in overestimation of the MICs of aminoglycosides and tetracyclines compared to caMHB (Table S1). The use of MHII for *F. tularensis* AST should be discouraged to avoid erroneous classification of some strains as resistant to aminoglycosides and/or tetracyclines using the CLSI breakpoints for susceptibility (concentration value threshold used for the categorization of a bacterial strain as susceptible, intermediately susceptible or resistant to an antibiotic). To date, antibiotic resistance reported in the literature for natural strains of *F. tularensis* have never been formally characterized by a reference laboratory. In the following paragraphs, the term resistant is applied to strain whose MICs are not classified as susceptible according to CLSI breakpoints for susceptibility, even though the mechanism of resistance has not been characterized. The MHII medium may also affect the activity of fluoroquinolones since ciprofloxacin MICs up to 0.25 mg/L were reported with this medium (Maurin et al., 2000; García del Blanco et al., 2004), while MICs for this antibiotic range from ≤ 0.002 to 0.125 mg/L (MIC_90_, ≤ 0.016–0.047 mg/L) for all other studies reported (Table 1 and Table S1).

Several agar media were used for MIC determination using the E-test strip technique (Tables 1 and Table S1). MIC ranges observed with these different solid media were similar to each other and to those obtained with the broth microdilution method using enriched caMHB. No strain was classified as resistant to any of the drugs used for tularaemia treatment and for which CLSI breakpoints have been defined (i.e., gentamicin, streptomycin, ciprofloxacin, levofloxacin, tetracycline, doxycycline and chloramphenicol). Thus, the *E*-test strip methodology appears to be a convenient alternative for antibiotic susceptibility categorisation of *F. tularensis* strains compared to the more time-consuming and fastidious broth microdilution method. Moreover, this method is less risky for laboratory personnel because it does not require handling large volumes of liquid suspensions of *F. tularensis*. However, no study has compared AST results using MIC strips to those of the microdilution reference method using enriched caMHB. Standardization of agar media for *F. tularensis* AST would be beneficial for comparison of studies conducted in different laboratories.

A few studies have compared *F. tularensis* antibiotic susceptibilities when using the agar dilution technique with different solid media (Table 1 and Table S1). Blood cysteine agar should be avoided since it identified resistant strains of *F. tularensis* for gentamicin or streptomycin, which was never confirmed by characterizing the resistance mechanisms involved. For the Thayer Martin agar and enriched Mueller-Hinton agar, results were concordant with those of the broth microdilution and MIC strip assays. One study compared MIC results using the agar dilution method, the MIC strip method or the broth microdilution reference method. Unfortunately, results obtained with the reference method were not reproducible. The correlation of results obtained with either the agar dilution or MIC strip tests, using enriched Mueller Hinton agar, were 87% for doxycycline, 94% for ciprofloxacin, but only 42% for gentamicin. The agar dilution method poorly correlated with the broth microdilution method, with only 72% correlation for doxycycline, 68% for ciprofloxacin and 51% for gentamicin.

According to our previous comments on MIC methods, we excluded MIC values obtained with the broth microdilution method using modified MHII broth and those determined by the agar dilution method using blood cystein agar plates (Table S1) MICs obtained with all other methods were recorded, although analyzed cautiously because of poor methodological standardization, as previously mentioned. The remaining 812 *F. tularensis* strains were categorized as susceptible to first-line antibiotics for tularaemia, including the aminoglycosides, the fluoroquinolones and the tetracyclines. MIC ranges and CLSI breakpoints for these antibiotics are shown in Table 2.

**Table 2:** **MIC and MIC_90_ ranges of aminoglycosides, tetracyclines and fluoroquinolones against *F. tularensis***.

**Antibiotics**	**MIC range (mg/L)**	**MIC_90_ range (mg/L)**	**CLSI breakpoint for susceptibility (mg/L)**
Gentamicin	≤0.016–2	0.064–1	≤ 4
Streptomycin	≤0.064–8	0.25–6	≤ 8
Tetracycline	≤0.094–2	≤ 0.25- 1	≤ 4
Doxycycline	0.064–4	0.25–2	≤ 4
Ciprofloxacin	≤0.002–0.125	≤ 0.016–0.064	≤ 0.5
Levofloxacin	≤0.004–0.125	0.012–0.125	≤ 0.5
Chloramphenicol	≤0.023–4	≤ 0.25–2	≤ 8

*Data are summarised from all studies selected for this review*.

#### Aminoglycosides, fluoroquinolones, and doxycycline

Altogether, among antibiotic classes recommended for first-line treatment of tularaemia, ciprofloxacin displayed the lowest ranges for MICs (≤ 0.002–0.125 mg/L) and MICs_90_ (≤ 0.016–0.064 mg/L) between studies. Gentamicin MICs ranged from ≤ 0.016 to 2 mg/L, and MICs_90_ from 0.064 to 1 mg/L. The MIC and MIC_90_ ranges for doxycycline were 0.064–4 mg/L and 0.25–2 mg/L, respectively (Table 1). Only slight differences in susceptibility to these antibiotics were observed between type A and type B strains of *F. tularensis*, or between various biovars or clades within type A and type B strains.

Among fluoroquinolones, ciprofloxacin and levofloxacin were the most active compounds *in vitro* (Table 1). All strains tested displayed MIC levels to these antibiotics at least 4-fold less than the CLSI breakpoint for susceptibility (Table 2). Moxifloxacin, norfloxacin, gatifloxacin, grepafloxacin, trovafloxacin and sparfloxacin were also effective against *F. tularensis in vitro* (Table 3), but the three latter antibiotics have been withdrawn from the market because of potential severe side effects.

**Table 3:** **MICs for the other aminoglycosides and fluoroquinolones evaluated against *F. tularensis***.

**Reference**	**Origin**	**Nb of strains Type/biovar**	**Tobramycin**		**Amikacin**		**Moxifloxacin**		**Norfloxacin**		**Grepafloxacin**		**Trovafloxacin**		**Gatifloxacin**		**Sparfloxacin**	
			**Range**	**MIC_90_**	**Range**	**MIC_90_**	**Range**	**MIC_90_**	**Range**	**MIC_90_**	**Range**	**MIC_90_**	**Range**	**MIC_90_**	**Range**	**MIC_90_**	**Range**	**MIC_90_**
**BROTH MICRODILUTION**																		
Georgi et al., 2012	Europe	69 B			≤ 0.5–2	2												
***E*****-TEST**																		
Yeşilyurt et al., 2011	Turkey	39 B biovar II	0.125–0.38	0.25	0.75–1	1	0.012–0.032	0.032										
Tomaso et al., 2005	Austria	50 B biovar II	0.19–3	0.75	1–16	4	0.016–0.19	0.125	0.023–0.19	0.125					0.008–0.047	0.032	0.003–0.047	0.023
Ikäheimo et al., 2000	Finland	38 B	0.5–2	1.5							0.016–0.047	0.047	0.012–0.047	0.032				
Johansson et al., 2002	USA	8 A					0.032–0.125				0.008–0.125				0.016–0.125		0.008–0.032	
		16 B					0.032–0.125	0.125			0.016–0.125	0.064			0.008–0.064	0.064	0.008–0.064	0.064

As for the aminoglycosides, tobramycin and amikacin were evaluated in three studies, only against type B strains (Ikäheimo et al., 2000; Tomaso et al., 2005; Yeşilyurt et al., 2011). Tobramycin displayed MIC values (0.125–3 mg/L) close to those of gentamicin and streptomycin. Interestingly, Enderlin et al., reported in 1994 a cure rate with this antibiotic of only 50% but for a limited number of patients (3/6 patients) (Enderlin et al., 1994). Conversely, amikacin was less active *in vitro*, with MICs up to 16 mg/L (Table 3; Tomaso et al., 2005).

Thus all strains were confirmed as susceptible to the first-line antibiotics recommended for tularemia treatment, with ciprofloxacin and levofloxacin showing the lowest MICs *in vitro*.

#### Macrolides

Erythromycin MICs are much higher for biovar II *F. tularensis* subsp. *holarctica* strains, than for biovar I strains of the same subspecies and for *F. tularensis* subsp. *tularensis* strains. Kudelina et al first described erythromycin-resistant strains of *F. tularensis* as those able to grow on media containing up to 6400 mg/L of this antibiotic, while susceptible strains could be killed by 25 mg/L of this antibiotic (Kudelina and Olsufiev, 1980). In the literature, type B biovar II strains, which currently correspond to East European strains belonging to the B12 subclade, were all resistant to erythromycin with MIC > 256 mg/L using the E-test strip method (Table 4; Karlsson et al., 2016). Only Tomaso et al. reported one biovar II strain with an erythromycin MIC of only 4 mg/L, but this result can be questioned according to current literature (Tomaso et al., 2005). Using the broth microdilution method, all type B biovar II strains had erythromycin MICs of ≥ 32 mg/L (Table 1). In contrast, all type B biovar I strains displayed erythromycin MICs ≤ 8 mg/L using the broth microdilution method and ≤ 1 mg/L using MIC strips. Among erythromycin-susceptible *F. tularensis* strains, subtle differences in erythromycin MICs could be found in the literature between the US strains belonging either to type B biovar I (MIC range, 0.125–2 mg/L; MIC_90_ range, 0.5–1 mg/L) or to type A (MIC range, 0.125–1 mg/L), and the West European type B biovar I strains (MIC range, 1–8 mg/L; MIC_90_, 4 mg/L). This difference might be correlated with the B6 clade displaying higher MIC levels, or to methodological differences, such as incubation of media in ambient air vs. under 5% CO_2_ atmosphere, the latter reducing the activity of the macrolides. The Japanese type B strains, including those formally identified as belonging to biovar japonica, displayed erythromycin MICs ranging from 0.094 to 1.5 mg/L.

**Table 4:** **MICs of tetracycline, tigecycline, macrolides (erythromycin, azithromycin) telithromycin, chloramphenicol, rifampicin and linezolid against *F. tularensis***.

**Reference**	**Origin**	**Nb of strains Type/biovar**	**Tetracycline**		**Tigecycline**		**Erythromycin**		**Azithromycin**		**Telithromycin**	**Chloramphenicol**		**Rifampicin**		**Linezolid**	
			**Range**	**MIC_90_**	**Range**	**MIC_90_**	**Range**	**MIC_90_**	**Range**	**MIC_90_**	**Range**	**Range**	**MIC_90_**	**Range**	**MIC_90_**	**Range**	**MIC_90_**
**BROTH MICRODILUTION**																	
Origgi et al., 2014	Switzerland	19 B6	≤ 0.25	≤ 0.25			2-8	4				≤ 2	≤ 2				
		5 B12	≤ 0.25				>32					≤ 2					
Georgi et al., 2012	Europe	69 B	≤ 0.125–2	1			1->16	>16				1–4	2	≤ 0.5–2			
	North America	7 A	≤ 0.125–0.5										≤ 0.5				
	Central Asia	4 ssp. *mediasiatica*	0.25–2									1–4					
Urich and Petersen, 2008	North America	92 A	0.25–2	1			0.5–4	2				0.5–4	2				
		77 B	0.12–2	1			0.5–2	0.5				0.5–4	2				
***E*****-TEST MIC STRIPS**																	
Johansson et al., 2000	Sweden	7 B					>256					0.25		0.5			
Hotta et al., 2013	Japan	36 B					0.094–1.5	1.5									
Kreizinger et al., 2013	Hungary	29 B12	0.19–0.72	0.5	0.094–0.19	0.19	>256	>256				0.5–1.5	1.5	0.5–2	1	12–48	32
Yeşilyurt et al., 2011	Turkey	39 B biovar II	0.125–0.5	0.38	0.094–0.38	0.25	>256	>256	>256	>256		0.094–0.25	0.25	0.25–1	0.75	0.5–2	1.5
Tomaso et al., 2005	Austria	50 B biovar II	0.125–0.75	0.75			4->256	>256				0.023–2	0.75	0.25–3	1.5		
Ikäheimo et al., 2000	Finland	38 B	0.094–0.5	0.38					>256	>256		0.125–0.5	0.38	0.094–0.38	0.25		
Kiliç et al., 2013	Turkey	249 B biovar II+ 1 biovar *japonica*	0.094–0.5	0.38			1->256	>256				0.094–0.75	0.5	0.125–1	0.75		
Johansson et al., 2002	USA	8 A					0.125–1		0.125–2			0.5–1		0.25–2		2–4	
		16 B					0.125–1	1	0.064–2	1		0.25–1	1	0.125–1	1	4–16	8
Velinov et al., 2011	Bulgaria	21 B biovar II					>256	>256				1–4	2				
**AGAR DILUTION**																	
Valade et al., 2008	France	71 B									0.125–0.25	0.25–2		0.015–0.5			
Johansson et al., 2000	Sweden	7B										0.5–1		1			

According to the above data, a breakpoint for the biovar II definition might be set at erythromycin MIC ≥ 32 mg/L, in agreement with Kudelina's above-mentioned study (Kudelina and Olsufiev, 1980). Moreover, erythromycin resistance in type B biovar II strains has recently been correlated with the presence of a single mutation A2059C in the three copies of the *rrl* gene, encoding the 23S rRNA (Karlsson et al., 2016). Thus, determination of the *rrl* gene sequence provides a confirmatory identification of biovar II subtype.

Azithromycin MICs against *F. tularensis* were also determined in three studies, with the same dichotomy between biovar I and biovar II of type B strains. MIC ranges were similar to those observed for erythromycin (Table 4). Biovar II strains displayed azithromycin MICs > 256 mg/L, whereas MIC ranges were 0.064–2 mg/L for 16 type B and 0.125–2 mg/L for eight type A strains from the USA (Ikäheimo et al., 2000; Johansson et al., 2002; Yeşilyurt et al., 2011). Telithromycin, a ketolide compound, displayed MICs ranging from 0.125 to 0.25 mg/L with the agar dilution method against type B biovar I strains (Valade et al., 2008).

Thus, low MIC values are observed for macrolides and one ketolide against biovar I strains. No susceptibility breakpoints are currently defined by the CLSI for these antibiotics against *F. tularensis*, although they may represent useful therapeutic alternatives for infection related to biovar I strains, especially if first-line antibiotics are contraindicated. Further experimental data in animal models are needed for the *in vivo* evaluation of the activity of these compounds against *F. tularensis*. In contrast, the macrolides and ketolides are ineffective against biovar II strains.

#### Beta-lactams

Many β-lactams have been tested *in vitro* against *F. tularensis*, although they are considered unreliable for treatment of tularaemia (Cross and Jacobs, 1993; Cross et al., 1995; Tarnvik, 2007; Maurin et al., 2011). In the literature, all the *F. tularensis* strains tested were resistant to penicillin A, ticarcillin and piperacillin, whether or not associated with a β-lactamase inhibitor, with MICs ≥ 64 mg/L (Table 5). In contrast, the cephalosporins, monobactams and carbapenems displayed larger MIC ranges. Although most *F. tularensis* strains displayed MICs higher than 32 mg/L to these β-lactams, a few strains displayed MICs lower than 1 mg/L (Table 5). In a study from Hotta et al., 30–60% of 36 Japanese strains displayed MICs lower than 1 mg/L for cefotaxime, ceftriaxone, cefoxitin, aztreonam, imipenem and meropenem (Hotta et al., 2013). Velinov et al. described 21 Bulgarian strains with a MIC range for ceftriaxone between 2 and 4 mg/L (Velinov et al., 2011). To date, two β-lactamase genes (*bla1* and *bla2*) have been identified in the LVS strain (with corresponding homologs in the Schu S4 strain), but the expression of recombinant LVS proteins in *Escherichia coli* only revealed the *bla2*_*LVS*_ gene to encode a functional β-lactamase (Bina et al., 2006). In 2012, Antunes et al. reported a class A β-lactamase (FTU-1) present in 14 strains belonging to all *F. tularensis* subspecies, including various type B strains from the USA, France, Japan, Russia, and Sweden (Antunes et al., 2012). Actually, the FTU-1 corresponds to the *bla2*_*LVS*_ gene, as evidenced by comparison of gene sequences (YP_513599.1 and FTT_0611c, respectively). This class A β-lactamase, which was partially inactivated by β-lactamase inhibitors, induced resistance to penicillin and a 4-fold increase in ceftazidime MIC when cloned and expressed in *E. coli*. This β-lactamase had no effect on other cephalosporins, monobactams and carbapenems (Bina et al., 2006; Antunes et al., 2012). The presence of the FTU-1/*bla2*_*LVS*_ gene is thus compatible with the cephalosporin-susceptibility phenotype observed in Japanese strains, whereas additional resistance mechanisms are probably involved in strains displaying resistance to cephalosporins, monobactams and carbapenems (Table 5). In 2008, Bina et al. characterized an AcrAB RND efflux system in the LVS strain, which conferred increased resistance to all beta-lactams tested in the study (i.e., ampicillin, carbenicillin and cefoperazone) but not to carbapenems (Bina et al., 2008). Thus, β-lactams are not recommended for the treatment of tularemia.

**Table 5:** **MICs of beta-lactams against *F. tularensis***.

**Reference**	**Origin**	**Nb of strains Type/biovar**	**MIC range (mg/L)**								
			**AMP**	**AMC**	**PIP/TAZ**	**CAZ**	**COX**	**FEP**	**IMI**	**MER**	**AZT**
**BROTH MICRODILUTION**											
Georgi et al., 2012	Europe	69 B		64->64		>32			16->16		
***E*-TEST**											
Hotta et al., 2013	Japan	34 B					0.047->256		0.047->32	0.094->32	0.75->256
Yeşilyurt et al., 2011	Turkey	39 B biovar II	>256	>256	>256	>256	>256	>256	>32		
Tomaso et al., 2005	Austria	50 B biovar II	>256		>256	>256	>32	>32	0.5->32	1.5->32	>256
Ikäheimo et al., 2000	Finland	38 B			>256	>256	>32		>32	>32	
Velinov et al., 2011	Bulgaria	21 B biovar II			>256		2-4				
**AGAR DILUTION**											
Scheel et al., 1993	Scandinavia	20 B				>32			>32	>32	>32

^*^ if available, MIC_90_ value corresponded every time to the highest value of MIC range. AMP, Ampicillin; AMC, Amoxicillin/Clavulanate; PIP/TAZ, Piperacillin/tazobactam; CAZ, Ceftazidime; COX, Ceftriaxone; FEP, Cefepime; IMI, Imipenem; MER, Meropenem; AZT, Aztreonam.

#### Chloramphenicol

The reported chloramphenicol MICs range from ≤ 0.023 to 4 mg/L, with an MIC_90_ range of 0.25–2 mg/L (Table 4). Chloramphenicol has been used occasionally (alone or in combination with other antibiotics) to treat patients with tularaemia meningitis, owing to its high distribution in brain tissue and cerebrospinal fluid (Tarnvik, 2007; Hofinger et al., 2009). Considering the MIC breakpoint of 8 mg/L, *F. tularensis* can be considered susceptible to chloramphenicol, but the clinical use of this antibiotic is currently restricted to meningitis because of potential severe bone marrow toxicity.

#### Rifampicin

Rifampicin is active *in vitro* against *F. tularensis* but with MICs ranging from ≤ 0.094 to 3 mg/L, and a MIC_90_ range of 0.25–1.5 mg/L (Table 4). However, this antibiotic is not recommended for tularaemia treatment because of potential rapid selection of resistant mutants, as described by Johansson et al. (2000) and Tarnvik (2007). It has been used in combination with ciprofloxacin for treatment of an infected total knee arthroplasty, with resolution of symptoms only after addition of rifampicin and a successful outcome with this antibiotic combination (Cooper et al., 1999).

#### New antibiotics

Among more recently developed antibiotics, tigecycline, a new glycylcycline, has been evaluated in two studies using the *E*-test method, with reported MICs (0.094–0.38 mg/L) and MIC_90_ (0.19–0.25 mg/L) ranges lower than those determined in the same studies for tetracycline and doxycycline (Tables 1, 4) (Yeşilyurt et al., 2011; Kreizinger et al., 2013). No breakpoints are currently defined for *F. tularensis* susceptibility to tigecycline, but all tested strains displayed MICs ≤ 0.5 mg/L. Further studies are needed to evaluate tigecycline MICs using the microdilution reference method. Importantly, fresh medium (<24 h) must be used to prevent overestimation of tigecycline MICs (Bradford et al., 2005). Tigecycline might be a suitable alternative to doxycycline for treatment of tularaemia, but its much broader antibacterial spectrum is a disadvantage due to a greater deleterious effect on the gut commensal flora.

Conflicting results were observed for linezolid, an oxazolidinone compound. The activity of this antibiotic is considered mainly restricted to Gram-positive bacteria, with a CLSI susceptibility breakpoint for the corresponding species set at ≤ 2 mg/L. However, MICs of 4–8 mg/L have been found for Gram-negative bacteria of the genera *Bacteroides, Moraxella* and *Pasteurella*, owing to the absence or low efficacy of efflux systems, or a high affinity of their ribosomes for linezolid (Livermore, 2003). As for *F. tularensis*, linezolid displayed MICs of 0.5–2 mg/L for 39 biovar II type B strains from Turkey, 2–4 mg/L for eight type A strains from the USA, and 4–16 mg/L for 16 type B strains from the USA, when using the *E*-test strip procedure. In contrast, MICs ranged from 12 to 48 mg/L for 29 Hungarian biovar II type B strains using the same technique, although such differences could be related to the use of different solid media for AST (Tables 1, 4). The relative susceptibility of *F. tularensis* to linezolid may be related to its small genome with a limited number of efflux systems, while MIC variations between strains may reflect variable expression of such efflux systems. This hypothesis needs to be further assessed on a larger panel of *F. tularensis* strains.

#### Conclusion

In conclusion, MIC data confirm that among the antibiotics recommended as first-line treatment of tularaemia, ciprofloxacin and levofloxacin display the lowest MIC ranges against *F. tularensis*. Gentamicin ranks second, while doxycycline has the highest MIC range with some strains displaying an MIC at 4 mg/L, which is the breakpoint for susceptibility (4 mg/L). However, no resistant strains to any of these antibiotics have been characterized so far. Moreover, chloramphenicol is active and may be used in combination for rare meningitis due to *F. tularensis*. Azithromycin and telithromycin may be useful alternatives for infection related to biovar I strains of *F. tularensis* subsp. *holarctica*, when all first line antibiotics are contraindicated, but still require confirmation of their efficacy in animal models. Tigecycline and rifampicin are active *in vitro* and should be also further evaluated in animal models. Rifampicin may be an interesting antibiotic in association to fluoroquinolones for rare bone and joint infections.

## MBCs and other bactericidal assays

A few *in vitro* studies have reported either MBC or time-kill kinetic experiments for *F. tularensis*. MBC testing is performed similarly to MIC testing using a broth microdilution method, but an aliquot of each well with no visible growth after 48 h incubation is plated on enriched chocolate agar media for CFU count determination. After 48 h incubation of the chocolate agar plates at 37°C, under 5% CO_2_ atmosphere, CFU counting can determine the MBC, which is the lowest antibiotic concentration allowing 3-log or greater reduction of the primary bacterial inoculum.

Time-kill kinetic experiments determine CFU counts over time in antibiotics containing cultures, incubated for 48 h at 37°C, under 5% CO_2_ atmosphere. Experiments are conducted using enriched caMHB medium, containing two, four or eight times the MIC of the tested antibiotic compound (Maurin et al., 2000; Caspar et al., 2014).

The MBCs of several antibiotics (ceftriaxone, streptomycin, amikacin, gentamicin, thiamphenicol, telithromycin, erythromycin, clarithromycin, rifampicin, ofloxacin, ciprofloxacin, pefloxacin, doxycycline, and cotrimoxazole) were determined against a single human strain of *F. tularensis* subsp. *holarctica* biovar I, using the modified MHII as the experimental medium (Maurin et al., 2000). MBCs were equal to MICs for ofloxacin and ciprofloxacin; 2-fold higher for the aminoglycosides (streptomycin, amikacin, and gentamicin), thiamphenicol, telithromycin, rifampicin, pefloxacin, and doxycycline; 4-fold higher for erythromycin; 16 times higher for cotrimoxazole; and 32 times higher for clarithromycin. Despite a MIC of 2 mg/L, a bactericidal effect was never observed with ceftriaxone against *F. tularensis*, although β-lactams are classically considered bactericidal drugs against most other bacteria susceptible to these compounds. The latter results reinforce prior statements that β-lactams and cotrimoxazole are not reliable for tularaemia treatment (Tarnvik, 2007). According to this work, most antibiotics tested could be considered as bactericidal against *F. tularensis*. However, it should be mentioned that the use of modified MH2 in these experiments may have influenced both the bacteriostatic and the bactericidal activity of antibiotics compared to caMHB, as previously discussed. Indeed, in that study, the MIC value for doxycycline was 8 mg/L, which is higher than usually found in other studies.

We recently evaluated MBCs for gentamicin and doxycycline against the LVS strain and two human strains of *F. tularensis* subsp. *holarctica* biovar I from France, using caMHB medium (Caspar et al., 2014). The MBC was 8-fold higher than the MIC for gentamicin (respectively, 2 and 0.25 mg/L) and could not be determined for doxycycline, which was mainly bacteriostatic (MIC = 0.25 mg/L) (Caspar et al., 2014). These data are more in agreement with the known bactericidal and bacteriostatic nature of these two antibiotics, respectively. Such a difference may participate to the lower relapse rates reported with gentamicin (Enderlin et al., 1994).

Finally, a third study measured the MBCs of ciprofloxacin, gentamicin and doxycycline against the LVS strain, using enriched caMHB medium (Aloni-Grinstein et al., 2015). The MBC/MIC ratios were measured at 1–2 for gentamicin and ≤ 4 for ciprofloxacin, confirming the bactericidal nature of these antibiotics against *F. tularensis*. The MBC/MIC ratio was equal to 4 for chloramphenicol. In contrast, the MBC/MIC ratio was ≥ 64 for doxycycline, demonstrating a bacteriostatic activity (Aloni-Grinstein et al., 2015).

In conclusion, MBC data investigated with suitable media for *F. tularensis* AST revealed a bactericidal activity for the aminoglycosides and the fluoroquinolones, but a bacteriostatic activity for doxycycline. These data should be further confirmed using a larger sample of *F. tularensis* strains.

## Cell systems

The activity of antibiotics against *F. tularensis* has rarely been evaluated in cell models. These models used either of the following cell systems: murine macrophage-like cell lines J774.1 or P388D1; kidney epithelial Vero cells from African green monkey; human cell lines, including lung epithelial A549 cells, pulmonary fibroblastic MRC5 cells, non-phagocytic kidney epithelial HEK 293 and THP-1 human monocytes; or human monocytes purified from buffy coat (Maurin et al., 2000; Golovliov et al., 2003; Madrid et al., 2013; Schmitt et al., 2013; Sutera et al., 2014). In these models, the multiplicity of infection (MOI, bacteria/eukaryotic cell ratios used for cell infection) varied between 10 and 3000 according to the *F. tularensis* strain used (i.e., *F. tularensis* subsp. *tularensis* SchuS4 strain, *F. tularensis* subsp. *holarctica* FSC200 strain, or LVS) and the nature of the eukaryotic cells. Cell infection usually occurs after 2–3 h of bacteria–cell contact. The penetration of bacteria within eukaryotic cells may be enhanced by centrifugation of infected cell monolayers (Maurin et al., 2000; Madrid et al., 2013; Schmitt et al., 2013; Sutera et al., 2014; Aloni-Grinstein et al., 2015). Then non-phagocytised bacteria are removed by adding gentamicin to the extracellular medium for 1–4 h, which is referred to as the gentamicin protection assay. The cell monolayers are then washed and incubated at 37°C in 5% CO_2_ atmosphere, either in drug-free medium (growth control) or in the presence of the tested antibiotics. At various incubation times, cell monolayers are washed and lysed with detergents, and CFU counts in cell lysates are determined. Antibiotic activity is deduced from the ratio of CFU counts in antibiotic-containing cultures compared to the drug-free growth control.

Alternative methods for the evaluation of antibiotic activity against intracellular *F. tularensis* have been proposed. A dye-uptake assay based on the capacity of eukaryotic cells to internalize a vital dye has recently been reported. In this model, activity of antibiotics is deduced from their ability to inhibit bacterial growth within eukaryotic cells, preventing lysis of the cell monolayers. The term “minimal inhibitory extracellular concentration” (MIEC) was coined to define the minimal extracellular antibiotic concentration capable of preventing cytotoxic effects of *F. tularensis* multiplication (Sutera et al., 2014). The turnaround time of the dye uptake assay was 2 days compared to 4–5 days for the CFU count-based model. Cell mortality could also be evaluated by measuring lactate dehydrogenase activity in cell supernatants (Madrid et al., 2013). More recently, a qPCR assay was used to determine *F. tularensis* growth in Vero cells (Aloni-Grinstein et al., 2015). MIECs were determined after 24 h infection, with results comparable to those obtained with the CFU method (Aloni-Grinstein et al., 2015). However, qPCR may overestimate viable bacterial loads since it quantifies DNA from both viable and non-viable bacteria.

### Fluoroquinolones

The intracellular activity of antibiotics against a French type B biovar I strain of *F. tularensis* (erythromycin MIC of 4 mg/L) was evaluated in a P388D1 murine macrophage-like cell model, using the CFU count methodology (Maurin et al., 2000). The results showed that ciprofloxacin and ofloxacin at 1 mg/L were the most potent compounds, with more than a 4- and 3-log reduction of bacterial inocula after only 48 h incubation of cultures, respectively (Maurin et al., 2000). In a systematic screening of FDA-approved drugs to identify compounds that may inhibit multiplication of biological threat agents, including *F. tularensis*, norfloxacin (50 μM, i.e., 16 mg/L) exhibited 83% protection of cells against cytotoxicity of the SchuS4 strain in J774.1 murine macrophages, as determined by the supernatant's lactate dehydrogenase activity (Madrid et al., 2013). In a dye-uptake assay using MRC5 cells, MIECs and MICs were equal for ciprofloxacin, levofloxacin and moxifloxacin, suggesting good penetration of these antibiotics within *F. tularensis*-infected cells (Sutera et al., 2014).

### Tetracyclines

In the P388D1 murine macrophage-like model, doxycycline at 4 mg/L only induced a 2-log reduction of bacterial inocula after 72 h incubation (Maurin et al., 2000). In the systematic screening assay of FDA-approved drugs, tetracycline completely protected infected cells from cytotoxicity of the SchuS4 strain at 50 μM (22 mg/L), and minocycline induced 92% survival of infected cells at 23 mg/L (Madrid et al., 2013). In the dye uptake assay, MIECs were also equal to MIC for doxycycline, also demonstrating good penetration within infected cells. However, MIEC values were eight times higher for doxycycline (0.5 mg/L) compared to ciprofloxacin (0.064 mg/L) against the two biovar I type B strains evaluated in the study. In another study, the same ciprofloxacin MIEC was found for the LVS strain in Vero cells, as determined by both qPCR and CFU count methods, but a lower MIEC (0.125–0.5 mg/L) was found for doxcycline (Aloni-Grinstein et al., 2015).

### Aminoglycosides

In the P388D1 murine macrophage-like cell model, gentamicin at 3 mg/L was not bactericidal after 48 h incubation of cultures and only induced a 1-log reduction of the bacterial inoculum after 72 h incubation. When tested at 10 mg/L, a 2-log reduction of bacterial counts was observed after 48 h incubation and more than 3-log reduction after 72 h (Maurin et al., 2000). In another study using the attenuated LVS strain and the Vero cell line, gentamicin at 20 mg/L did not show any intracellular activity after 32 h incubation (Aloni-Grinstein et al., 2015). In the dye-uptake assay using MRC5 cells, the gentamicin MIEC after 48 h incubation of cultures was 2 mg/L against two French type B biovar I strains of *F. tularensis*, while the MICs of this antibiotic were, respectively, 0.25 and 0.5 mg/L for the same strains (Sutera et al., 2014). Delayed activity of gentamicin correlated with the slow penetration of this antibiotic within eukaryotic cells, usually detectable only after 48–72 h of antibiotic–cell contact.

The other aminoglycosides also penetrate slowly within eukaryotic cells. Amikacin and streptomycin, at 10 mg/L, only decreased *F. tularensis* inoculum by 1 log after 48 h, and 2 logs after 72 h (Maurin et al., 2000). In contrast, netilmicin did not prevent cell lysis in J774.1 cells infected with the SchuS4 strain (Madrid et al., 2013).

### Macrolides/lincosamides/ketolides

Conflicting results have been reported between studies testing the intracellular form of *F. tularensis*, which may be due to the use of different cell types and bacterial strains. In P388D1 murine macrophage-like cells, erythromycin and clarithromycin at 4 mg/L exhibited no intracellular activity against a type B biovar I strain of *F. tularensis* displaying MICs of 4 and 8 mg/L in acellular media for these antibiotics, respectively. In contrast, telithromycin at 4 mg/L (MIC at 0.5 mg/L in acellular medium) was able to reduce the bacterial inoculum between two to three log_10_ after 72 h (Maurin et al., 2000). Unlike telithromycin, the intracellular concentration of erythromycin and clarithromycin was probably not sufficient in this experiment to kill bacteria, as the extracellular concentration used was equal or superior to their MIC. In another study, a high concentration of erythromycin (50 μM, 37 mg/L) conferred full protection of J774.1 cell viability after infection with the SchuS4 type A strain (Madrid et al., 2013). In the same assay, clindamycin (50 μM, 21 mg/L) exhibited 83% protection of cells against the cytotoxic effect of *F. tularensis* proliferation (Madrid et al., 2013). In the dye uptake assay reported by Sutera et al., erythromycin MIECs were four times lower than MICs (respectively, 1–2 mg/L and 4–16 mg/L) for two French biovar I type B strains in MRC5 cells (Sutera et al., 2014). Altogether, these experiments show that erythromycin may be effective in preventing intracellular replication of type A and biovar I type B strains.

In another experiment reported by Ahmad et al., azithromycin (a C15 macrolide) showed complete killing of the LVS strain at 5 mg/L in murine macrophage J774.1 cells, although this antibiotic displays an MIC of 25 mg/L against this biovar II type B strain (Ahmad et al., 2010). This result suggests a high bactericidal concentration of azithromycin within *F. tularensis*-infected J774.1 macrophages. This may not be true for all cell types since a previous experiment from Ahmad et al., demonstrated that azithromycin was fully bactericidal at only 25 mg/L against the LVS strain grown in human lung epithelial A549 cells (Ahmad et al., 2010).

### Beta-lactams

In the P388D1 murine macrophage-like model, β-lactams at 10 mg/L (penicillin G, amoxicillin, ceftriaxone) displayed no activity against intracellular *F. tularensis* (Maurin et al., 2000). Meropenem was ineffective against both intracellular and extracellular *F. tularensis* in the dye uptake assay (Sutera et al., 2014).

### Other antibiotics

In the P388D1 murine macrophage-like model, thiamphenicol at 4 mg/L displayed no intracellular activity. In contrast, rifampicin at 4 mg/L induced a 2-log reduction of bacterial inocula after 72 h incubation (Maurin et al., 2000). In the dye uptake assay using MRC5 cells, MIECs were close to MICs for rifampicin, also suggesting good penetration of this compound within *F. tularensis*-infected cells. However, MIEC values were 16 times higher than those of ciprofloxacin (1 vs. 0.064 mg/L, respectively) against the two biovar I type B strains tested for *F. tularensis* (Sutera et al., 2014). Interestingly, linezolid exhibited higher activity against intracellular than extracellular *F. tularensis*, suggesting its accumulation within MRC5 cells, with a MIEC of 1 mg/L and an MIC of 8 mg/L (Sutera et al., 2014). Finally, daptomycin was ineffective both intracellularly and extracellularly (Sutera et al., 2014).

### Conclusion of intracellular activity of antibiotics against tularaemia

To summarize the data from the intracellular models, the fluoroquinolones ciprofloxacin, levofloxacin, moxifloxacin and ofloxacin displayed the lowest MIECs and the fastest bactericidal activity against intracellular *F. tularensis*, suggesting rapid and efficient concentration of these compounds within infected eukaryotic cells. The tetracyclines were less effective and mainly bacteriostatic against intracellular *F. tularensis*. The aminoglycosides displayed a bactericidal activity, but only after 48–72 h of antibiotic exposure of infected cells, which correlated with the slow intracellular accumulation of these basic compounds (Maurin and Raoult, 2001). Thus, the aminoglycosides are probably much more effective against the extracellular form of *F. tularensis* at the early stage of antibiotic treatment, while these antibiotics may also be active against intracellular *F. tularensis* after several days of treatment. Streptomycin and amikacin were slightly less effective than gentamicin at the same concentration, and netilmicin was ineffective. The beta-lactams and daptomycin were not effective in cell systems. In contrast, the activity of linezolid against intracellular *F. tularensis* should be further investigated both *in vitro* and *in vivo*.

Interestingly, in two studies, the macrolides erythromycin and azithromycin were found to be active against the intracellular form of type A and biovar I type B *F. tularensis* strains, which are naturally susceptible to these antibiotics. Azithromycin was also effective against the intracellular form of biovar II type B LVS strain, although naturally more resistant to macrolides, but only in phagocytic and fibroblastic cells. Indeed, the intracellular accumulation of the macrolides varies according to the eukaryotic cell type considered, with a lower accumulation in epithelial cells compared to phagocytic and fibroblast cells (Ahmad et al., 2010; Sutera et al., 2014). PK/PD studies have demonstrated the accumulation of azithromycin within human phagocytic and fibroblast cells, and in many human tissues, such as lungs, soft tissues, prostate and tonsils (McDonald and Pruul, 1991; Matzneller et al., 2013). The intracellular/extracellular azithromycin ratio in tissues is generally between 10 and 100, and can be >200 in polymorphonuclear (PMN) leukocytes, with probable large amounts of this antibiotic at the site of infection (Hand and Hand, 2001; Hall et al., 2002; Matzneller et al., 2013). The intracellular accumulation of erythromycin is lower, with intracellular/extracellular ratios generally between 1 and 10 in tissues and PMNs (McDonald and Pruul, 1991; Hand and Hand, 2001). The activity of azithromycin against intracellular *F. tularensis* warrants further evaluation of its activity in animal models. These experiments should be conducted using type A and type B strains, especially the type B biovar II strains.

## Animal models

Although several animal models have been developed to study the pathogenesis of *F. tularensis* infection and vaccine efficacy, few of these models have evaluated *in vivo* antibiotic efficacy (Rick Lyons and Wu, 2007). This may be related to the absence of an optimal animal model mimicking human infection. In these models, antibiotic activity is usually deduced from the death rate and survival time of infected animals and the bacterial load in their organs (especially in the spleen) at the time of death or sacrifice, when treated with various antibiotic regimens compared to infected and untreated controls. BALB/c and C57BL/6 mice and guinea pigs are highly susceptible hosts to *F. tularensis* infection, but also to the LVS strain of *F. tularensis* subsp. *holarctica* and *F. novicida* strains, which have often been used as surrogates of *F. tularensis*, although they are much less virulent in humans (Stundick et al., 2013; Kingry and Petersen, 2014). Rabbits and Fisher 344 rats are less susceptible to *F. tularensis* infection and may better mimic human infection. However, variable immune responses to *F. tularensis* infection also exist between rat strains, with Sprague-Dawley being much more resistant than Fisher 344 rats (Raymond and Conlan, 2009). Results may also vary according to the route of infection, i.e., the intraperitoneal, intradermal, subcutaneous or intranasal routes for Fischer 344 rats (Stundick et al., 2013). Moreover, the “Animal Rule” states that experimental animal models should be developed using the true etiologic agent causing human disease. Thus, evaluation of antibiotic activity in Fisher 344 rats infected with virulent type A or type B strains would be more predictive of the clinical situation.

Few experiments have evaluated the activity of antibiotics in *F. tularensis*-infected animal models. However, interesting data have been obtained regarding the *in vivo* efficacy of antibiotics, and relapse rates according to delay in antibiotic therapy and treatment duration. Unfortunately, these models are highly heterogeneous with respect to the route of infection, the route of antibiotic administration, the antibiotic dose administrated and the *F. tularensis* strain tested.

### Fluoroquinolones and doxycycline

In 1998, Russel et al evaluated the effect of subcutaneous injection of doxycycline or ciprofloxacin on the median lethal dose (MLD) of the Schu S4 strain injected intraperitoneally to Porton outbred mice (Russell et al., 1998). The animals either received a 2-day antibiotic prophylaxis before the bacterial challenge, or were treated 48 h post-infection with 20 or 40 mg/kg twice a day, of doxycycline or ciprofloxacin for 10 days, and then were monitored for 24 days. Although the MLD was close to 1 CFU of the Schu S4 strain in untreated mice, full protection at the highest bacterial inoculum tested (8.8 × 10^6^ CFU) was achieved by a 2-day prophylaxis with ciprofloxacin or doxycycline, and by 10 days of post-infection treatment with either of these antibiotics at 40 mg/kg twice a day. At this dosage, the serum concentrations were higher than 4 × MIC for at least 12 h for ciprofloxacin, and 3 × MIC for 9 h for doxycycline (Russell et al., 1998).

The *in vivo* efficacy of fluoroquinolones was confirmed in 2005, by Piercy et al. (2005). BALB/c mice were infected subcutaneously with 10^6^ CFU of the Schu S4 strain and treated orally with 100 mg/kg of ciprofloxacin, gatifloxacin or moxifloxacin, from 6 h to 14 days post-infection. Survival rates at day 42 post-infection were 94, 100, and 100%, respectively, for these three antibiotics (Piercy et al., 2005).

In 2008, Klimpel et al. showed that a 13-day course of levofloxacin (5 mg/kg/day, intraperitoneally), starting 24 h following an intranasal challenge with 3 LD_50_ of *F. tularensis* Schu S4 strain, prevented death of all Balb/c mice (Klimpel et al., 2008). No bacteria were detectable in the spleen of the animals after 10 days of treatment, while very few were detected in the lungs at this time (Klimpel et al., 2008).

In 2012, Rotem et al. compared the efficacy of ciprofloxacin and doxycycline in Balb/c mice infected by inhalation of a 100-LD_50_ dose of *F. tularensis* LVS (10^5^ CFU) or Schu S4 (10^2^ CFU) strains (Rotem et al., 2012). Untreated controls died 5–7 days post-infection, whereas all LVS-infected mice were cured by intraperitoneal injection of ciprofloxacin (50 mg/kg bid) for 7 days or doxycycline (40 mg/kg bid) for 14 days, whether the antibiotic treatment was started at 24, 48, or 72 h post-infection (Rotem et al., 2012). *F. tularensis* LVS strain was undetectable by culture in the lungs, liver and spleen after completion of any of these treatments. In contrast, for animals infected with the Schu S4 strain, no death occurred when ciprofloxacin was administered 24 or 48 h post infection, while doxycycline only cured 90% of the animals even when administrated 24 h post-infection (Rotem et al., 2012).

### Treatment delay

In the study from Russel et al., a 48-h prophylaxis with ciprofloxacin or doxycycline protected mice from a 3.7 × 10^6^ CFU and 6.0 × 10^6^ CFU challenge with the Schu S4 strain, respectively (Russell et al., 1998). In contrast, when antibiotic treatment was started 24 h post-infection, complete protection was only obtained against an intraperitoneal challenge with 880 CFU for ciprofloxacin and 100 CFU for doxycycline. Thus, antibiotic efficacy was dramatically reduced after only 24 h infection (Russell et al., 1998). In the study by Rotem et al., in which mice were infected via Schu S4 strain aerosols, no death occurred when ciprofloxacin was administered 24–48 h post infection, while a 30% mortality rate was observed when this antibiotic was administered 3 days post-infection (Rotem et al., 2012). Doxycycline only cured 90, 30, and 0% of the mice when started at 24, 48, and 72 h post-infection, respectively (Rotem et al., 2012). In the study reported by Piercy et al., the survival rates decreased dramatically (94, 67, and 0%) when ciprofloxacin was administrated 6, 24, or 48 h post-infection, respectively (Piercy et al., 2005). In contrast, the survival rates were 100, 96, and 84% for gatifloxacin, and 100, 100, and 62% for moxifloxacin, respectively, showing the better therapeutic efficacy of these drugs compared to ciprofloxacin. In this study, the infectious dose was much higher than in that from Rotem et al (10^6^ CFU intraperitoneally vs. 10^2^ CFU intranasally). Finally, in the Klimpel et al. study, full protection against the SchuS4 strain (99 CFU intraperitoneally) was observed for levofloxacin at 40 mg/kg/day, as long as the treatment delay did not exceed 72 h (Klimpel et al., 2008). If started at 72 h, bacterial load decreased approximately from 10^6^ to 10 CFU per organ after 1 week of treatment. Delaying the treatment of more than 120 h resulted in the death of all infected mice (Klimpel et al., 2008).

### Bactericidal activity and treatment duration

In the Russel et al. study, when treatment was administrated for 5 days rather than 10 days post-infection, the MLD was reduced from > 8.8 × 10^6^ CFU to 3.7 × 10^6^ CFU for ciprofloxacin and 6.0 × 10^6^ CFU for doxycycline (Russell et al., 1998). The protective effect of the antibiotic therapy also decreased when animals were treated with a lower dose (20 mg/kg bd rather than 40 mg/kg bd) of ciprofloxacin or doxycycline. Moreover, the authors reported that death occurred in 90% of animals after antibiotic treatment withdrawal, demonstrating that a 5-day course of antibiotic treatment was not sufficient to eradicate *F. tularensis* (Russell et al., 1998). Thus, in this study, the dose and length of the antibiotic therapy administered highly influenced the outcome of *F. tularensis* infection in animals (Russell et al., 1998).

Significant insight into relapses came from the study reported by Piercy et al. (2005). Among the nine groups tested (ciprofloxacin, moxifloxacin or gatifloxacin, initiated 6, 24, or 48 h post-infection), these authors showed that the administration of 7 days of dexamethasone to surviving mice in order to abolish their immune system resulted in 17–73% relapse rates depending on the treatment group. These findings suggested that quiescent bacteria controlled by the immune system persisted in mice despite 14 days of fluoroquinolone therapy. Fluoroquinolones were thus not fully bactericidal when a high inoculum of *F. tularensis* was injected to the mice (e.g., subcutaneous injection of 10^6^ CFU of the Schu S4 strain). Suppression of the immune system enabled latent bacteria to multiply and kill mice, even in the 6-h post-exposure treatment group (respectively, 36 and 42% mortality rates in the ciprofloxacin- and moxifloxacin-treated groups; Piercy et al., 2005).

Data from the study by Rotem et al. corroborated this hypothesis (Rotem et al., 2012). In their experiments with the virulent Schu S4 strain, no death occurred when ciprofloxacin was administered 24 or 48 h post-infection, while a 30% mortality rate was observed when this antibiotic was administered 3 days post-infection. In the latter case, bacteria were undetectable in organs at the time of antibiotic treatment withdrawal, while cultures were positive 3 days later for three out of 10 mice that died after antibiotic withdrawal. The extension of ciprofloxacin treatment duration from 7 to 10 days cured all animals, even when the antibiotic was administrated 72 h post-infection. In contrast, when doxycycline was started 72 h post-infection, all animals had approximately 100 CFU/g of tissue (lungs, liver or spleen) at the end of treatment, after which the bacterial loads in organs increased in all mice until death. Extension of treatment from 14 to 21 days resulted in no cultivable bacteria in lungs, liver or spleen at the end of treatment, but all mice relapsed after 2 days of doxycycline withdrawal (Rotem et al., 2012). A different picture was observed with the LVS strain, which was below detectable level by culture in the lungs, liver and spleen after completion of any of the treatment regimens (ciprofloxacin for 7 days or doxycycline for 14 days, started 24, 48, and 72 h post-infection) in the mice sacrificed at the end of the treatment. However, 2 days after doxycycline treatment withdrawal, the LVS strain was detected in all organs, and bacterial loads gradually decreased to an undetectable level in the following 7 days and resulted in no death. Thus, although undetectable after doxycycline treatment, the attenuated LVS strain was not fully eradicated and regrowth of the bacteria was observed as soon as doxycycline was stopped, although it did not kill the mice. In contrast, re-emergence of bacteria did not occur for the ciprofloxacin group of LVS-infected mice after ciprofloxacin withdrawal.

### Conclusions drawn from animal model data

Altogether, these data indicate that both ciprofloxacin and doxycycline are able to prevent tularaemia in mice when treatment is started 48 h before a challenge with *F. tularensis* SchuS4 strain. These antibiotics are also able to cure the disease and eradicate *F. tularensis* when administrated post-infection at a concentration of 40 mg/kg or higher, for 5–10 days. Efficacy of the antibiotic treatment is highly correlated with the antibiotic dose administered, which is probably related to the duration over which serum concentrations of antibiotics are above the MIC values of the infecting strain.

However, when treatment was started 24–72 h post-infection, ciprofloxacin was superior to doxycycline: (1) in the study conducted by Rotem et al., doxycycline was not able to cure all mice when infected with 10^2^ CFU of the SchuS4 strain via the aerosol route, even when started at 24 h post-infection, while ciprofloxacin cured all mice when started either 24 or 48 h post-infection (Rotem et al., 2012); (2) relapse rates were higher for doxycycline as it was unable to fully eradicate the Schu S4 or LVS strains from the lungs, liver and spleen, even when administrated for 14–21 days; (3) the time until antibiotic treatment initiation reduced doxycycline efficacy much more than for ciprofloxacin.

*F. tularensis* multiplies within the slightly acidic cytosol of eukaryotic cells, and a low pH may alter the activity of the basophilic antibiotics ciprofloxacin and doxycycline (Russell et al., 1998). Data obtained in animal models indicate that persistent bacteria may develop in hosts infected with *F. tularensis* when the antibiotic therapy administered has poor bactericidal activity or is too short in duration. These data suggest that long antibiotic treatment duration should be used in immunocompromised patients infected with *F. tularensis*. This could also be true for patients suffering from suppurated lymphadenopathy, who often experience relapses despite administration of ciprofloxacin for 14 days or doxycycline for 21 days.

Treatment must be initiated as soon as possible since antibiotic efficacy decreased significantly when treatment was delayed by 24–48 h post-infection. It should be noted that, because of diagnosis delays, antibiotics are often given several days to weeks after infection in patients suffering from tularaemia. Importantly, moxifloxacin efficacy was less impacted by treatment delay than ciprofloxacin and thus may represent an advantageous therapeutic option in case of late diagnosis. MIC values are close for moxifloxacin and ciprofloxacin.

## General conclusion and perspectives

Since the introduction of streptomycin in the mid-1950s, antibiotic treatment of human tularaemia has remained challenging. In most tularaemia endemic countries, human infections are now rare, and *F. tularensis* isolation is even rarer. Thus, AST data for isolated strains of *F. tularensis* may not reflect the true situation of current antibiotic resistance in this pathogen. Importantly, the analysis of AST methodological variations between studies shows an urgent need for international standardization. Following the CLSI guidelines, including using the appropriate experimental medium, bacterial inoculum and incubation time are mandatory, although some *F. tularensis* strains grow better or exclusively under 5% CO_2_ atmosphere. The broth microdilution technique using enriched caMHB medium should be considered the reference method. As recommended by the CLSI, a final inoculum calibrated at 5 10^5^ CFU/mL should be used, as a 10-fold higher or lower inoculum is associated to interpretation errors (Georgi et al., 2012). However, the modified Mueller-Hinton II liquid medium should not be used for *F. tularensis* AST to avoid reporting MICs that could categorize *F. tularensis* strains as resistant to aminoglycosides or doxycycline whereas such resistances have never been characterized so far in this pathogen. From the data currently available, use of the CLSI broth microdilution method can be recommended to test large collections of *F. tularensis* strains in reference laboratories, while the E-test method would be more convenient for testing one or a few strains. However, the E-test MIC strip method, although more convenient to perform, still requires comparative studies with the reference method.

Altogether, available AST data indicate that the fluoroquinolones display the lowest MICs, are strongly and rapidly bactericidal in cell models and are highly effective in curing *F. tularensis* infection in infected mice when administered for at least 10 days. Among the fluoroquinolones, ciprofloxacin and levofloxacin have the lowest MICs. In contrast, doxycycline MICs are closer to the CLSI susceptibility breakpoint and this antibiotic is only bacteriostatic in cell models. In the mouse model, a 14-day course of doxycycline did not eradicate *F. tularensis* when treatment was started 72 h post-infection (Rotem et al., 2012). A 21-day course of treatment was still not fully effective to eradicate bacteria, whereas 10 days of ciprofloxacin treatment was effective. Therefore, doxycycline should be considered less effective than fluoroquinolones for treatment of tularaemia, which is consistent with higher therapeutic failure rates in humans observed with this antibiotic. Because antibiotic treatment is often administrated several days after symptom onset in tularaemia patients, ciprofloxacin may be a better choice than doxycycline. Moreover, treatment duration with ciprofloxacin of at least 14 days should be recommended, while 3 weeks would be a minimum for doxycycline (Rotem et al., 2012). A longer duration of antibiotic treatment should probably be considered in case treatment is delayed longer than 2 weeks after symptom onset, especially if lymph node suppuration or other local or systemic complications have occurred. The aminoglycosides (especially gentamicin) also have low MICs. They are rapidly bactericidal in acellular media, but their intracellular bactericidal activity only takes place after 72 h of cell exposure owing to their slow penetration through the eukaryotic cell membrane. In the 1994 review by Enderlin et al., streptomycin was considered the most reliable treatment for human tularaemia, with almost no relapse after treatment removal (Enderlin et al., 1994). The aminoglycosides are still considered the most reliable treatment of severe tularaemia cases (e.g., the pneumonic and typhoidal forms of tularaemia, and all other systemic infections). Experimental data suggest that the combination of an aminoglycoside with a fluoroquinolone may currently be the most effective alternative in patients with severe tularaemia, especially to obtain a rapid bactericidal activity against extracellular and intracellular *F. tularensis*. However, one has always to remember that antimicrobial potency deduced from MIC values, MBC values, *in vitro* and *in vivo* bactericidal activity in animal models is only one of many factors (including pharmacokinetic and pharmacodynamic parameters, tolerability, plasma protein binding, tissue penetration, bactericidal activity, and contraindication) that may influence the decision of what drug to use in a clinical setting.

The macrolides (especially azithromycin) could be an alternative in patients infected with erythromycin-susceptible strains of *F. tularensis* (type A and type B biovar I) and in those for whom the above first-line treatments are contraindicated, including young children and pregnant women. However, the macrolide-resistant type B biovar II strains are currently found in Eastern Europe and in Asia, and co-exist with biovar I strains in Germany, Switzerland and Scandinavia. The empirical use of a macrolide in tularaemia patients cannot be considered reliable in these areas. The use of a macrolide in patients with mild to moderate severity tularaemia is safer in Western Europe, where only type B biovar I strains cause human infections. In this context, quick discrimination between type B biovar I and II strains either by determination of erythromycin susceptibility (using MIC strips or sequencing of *rrl* gene) or by detection of particular genetic markers (detection of RD23 deletion only found in West European strains) would be of particular interest (Dempsey et al., 2007; Pilo et al., 2009; Karlsson et al., 2016). Since these antibiotics mainly display a bacteriostatic activity against *F. tularensis*, they cannot be considered reliable to treat severe forms of tularaemia. Azithromycin combined with lymph node resection proved to be effective to cure a pregnant women suffering from oropharyngeal tularaemia with no complications for the fetus (Dentan et al., 2013). Further *in vivo* data in animal models and humans are needed before azithromycin can be recommended as a reliable alternative for treatment of tularaemia.

Finally, the causes of the high relapse rates observed in tularaemia patients after administration of a fluoroquinolone or a tetracycline remain undetermined. Several hypotheses can be proposed, including low penetration of antibiotics in tissues and eukaryotic cells *in vivo*; low susceptibility of bacteria *in vivo* especially because of low replication and persistence; inactivation of antibiotic activity *in vivo* owing to local conditions (e.g., acidic pH, enzymatic inactivation, etc.) and acquired resistance to antibiotics *in vivo* in patients under fluoroquinolone or tetracycline treatment. Further evaluation of such hypotheses in *in vitro* and *in vivo* experimental models is warranted.

## Author contributions

YC has collected data on antibiotic susceptibility testing (AST) of *Francisella tularensis*, both *in vitro* and in animal models, from the English and French literature in the PubMed database, since the introduction of fluoroquinolones in the treatment of tularaemia in 1989 until December 2016. He has written the article. MM has participated to data analysis and corrected the text of the article.

## Funding

The French National Reference Center for Tularemia is funded by Santé Publique France, the French Agency for Public Health.

### Conflict of interest statement

The authors declare that the research was conducted in the absence of any commercial or financial relationships that could be construed as a potential conflict of interest.

## References

[B1] Abd H. Johansson T. Golovliov I. Sandström G. Forsman M. (2003). Survival and growth of *Francisella tularensis* in *Acanthamoeba castellanii*. Appl. Environ. Microbiol. 69, 600–606. 10.1128/AEM.69.1.600-606.2003 12514047 PMC152416

[B2] Ahmad S. Hunter L. Qin A. Mann B. J. van Hoek M. L. (2010). Azithromycin effectiveness against intracellular infections of Francisella. BMC Microbiol. 10, 123. 10.1186/1471-2180-10-123 20416090 PMC2881020

[B3] Akalın H. Helvacı S. Gedikoğlu S. (2009). Re-emergence of tularemia in Turkey. Int. J. Infect. Dis. 13, 547–551. 10.1016/j.ijid.2008.09.020 19119037

[B4] Aloni-Grinstein R. Shifman O. Lazar S. Steinberger-Levy I. Maoz S. Ber R. (2015). A rapid real-time quantitative PCR assay to determine the minimal inhibitory extracellular concentration of antibiotics against an intracellular *Francisella tularensis* Live Vaccine Strain. Front. Microbiol. 6:1213. 10.3389/fmicb.2015.01213 26579112 PMC4630301

[B5] Antunes N. T. Frase H. Toth M. Vakulenko S. B. (2012). The class A β-Lactamase FTU-1 is native to *Francisella tularensis*. Antimicrob. Agents Chemother. 56, 666–671. 10.1128/AAC.05305-11 22083489 PMC3264220

[B6] Baker C. N. Hollis D. G. Thornsberry C. (1985). Antimicrobial susceptibility testing of *Francisella tularensis* with a modified Mueller-Hinton broth. J. Clin. Microbiol. 22, 212–215. 4031036 10.1128/jcm.22.2.212-215.1985 PMC268361

[B7] Barut S. Cetin I. (2009). A tularemia outbreak in an extended family in Tokat Province, Turkey: observing the attack rate of tularemia. Int. J. Infect. Dis. 13, 745–748. 10.1016/j.ijid.2008.12.002 19195917

[B8] Bicakci Z. Parlak M. (2008). A neglected cause of cervical lymphadenitis. Oropharyngeal tularemia. Saudi Med. J. 29, 1059–1061. 18626545

[B9] Bina X. R. Lavine C. L. Miller M. A. Bina J. E. (2008). The AcrAB RND efflux system from the live vaccine strain of *Francisella tularensis* is a multiple drug efflux system that is required for virulence in mice. FEMS Microbiol. Lett. 279, 226–233. 10.1111/j.1574-6968.2007.01033.x 18179581

[B10] Bina X. R. Wang C. Miller M. A. Bina J. E. (2006). The Bla2 β-lactamase from the live-vaccine strain of *Francisella tularensis* encodes a functional protein that is only active against penicillin-class β-lactam antibiotics. Arch. Microbiol. 186, 219–228. 10.1007/s00203-006-0140-6 16841206

[B11] Bradford P. A. Petersen P. J. Young M. Jones C. H. Tischler M. O'Connell J. (2005). Tigecycline MIC testing by broth dilution requires use of fresh medium or addition of the biocatalytic oxygen-reducing reagent oxyrase to standardize the test method. Antimicrob. Agents Chemother. 49, 3903–3909. 10.1128/AAC.49.9.3903-3909.2005 16127069 PMC1195415

[B12] Broman T. Thelaus J. Andersson A.-C. Backman S. Wikstrom P. Larsson E.. (2011). Molecular detection of persistent *Francisella tularensis* Subspecies *holarctica* in natural waters. Int. J. Microbiol. 2011:851946. 10.1155/2011/851946 20885922 PMC2946586

[B13] Busse H.-J. Huber B. Anda P. Escudero R. Scholz H. C. Seibold E.. (2010). Objections to the transfer of *Francisella novicida* to the subspecies rank of *Francisella tularensis*-response to Johansson et al. Int. J. Syst. Evol. Microbiol. 60, 1718–1720. 10.1099/00207713-60-8-1718 20688749

[B14] Caspar Y. Sutera V. Boisset S. Denis J.-N. Maurin M. (2014). Bis-indolic compounds as potential new therapeutic alternatives for tularaemia. Front. Cell. Infect. Microbiol. 4:24. 10.3389/fcimb.2014.00024 24579066 PMC3936198

[B15] Celebi G. Baruönü F. Ayoğlu F. Cinar F. Karadenizli A. Uğur M. B.. (2006). Tularemia, a reemerging disease in northwest Turkey: epidemiological investigation and evaluation of treatment responses. Jpn. J. Infect. Dis. 59, 229–234. 16936340

[B16] Cerný Z. (2001). Changes of the epidemiology and the clinical picture of tularemia in Southern Moravia (the Czech Republic) during the period 1936-1999. Eur. J. Epidemiol. 17, 637–642. 10.1023/A:1015551213151 12086077

[B17] Chanturia G. Birdsell D. N. Kekelidze M. Zhgenti E. Babuadze G. Tsertsvadze N.. (2011). Phylogeography of *Francisella tularensis* subspecies holarctica from the country of Georgia. BMC Microbiol. 11:139. 10.1186/1471-2180-11-139 21682874 PMC3224097

[B18] Christova I. Velinov T. Kantardjiev T. Galev A. (2004). Tularaemia outbreak in Bulgaria. Scand. J. Infect. Dis. 36, 785–789. 10.1080/00365540410021199 15764161

[B19] Clemens D. L. Horwitz M. A. (2007). Uptake and intracellular fate of *Francisella tularensis* in human macrophages. Ann. N. Y. Acad. Sci. 1105, 160–186. 10.1196/annals.1409.001 17435118

[B20] CLSI. M45-A2 (2010). Methods for Antimicrobial Dilution and Disk Susceptibility Testing of Infrequently Isolated or Fastidious Bacteria; Approved Guideline-Second Edition. M45A2. Wayne.

[B21] Cooper C. L. Caeseele P. V. Canvin J. Nicolle L. E. (1999). Chronic prosthetic device infection with *Francisella tularensis*. Clin. Infect. Dis. 29, 1589–1591. 10.1086/313550 10585830

[B22] Cross J. T. Jacobs R. F. (1993). Tularemia: treatment failures with outpatient use of ceftriaxone. Clin. Infect. Dis. 17, 976–980. 10.1093/clinids/17.6.976 8110955

[B23] Cross J. T. Schutze G. E. Jacobs R. F. (1995). Treatment of tularemia with gentamicin in pediatric patients. Pediatr. Infect. Dis. J. 14, 151–152. 10.1097/00006454-199502000-00014 7746700

[B24] Dempsey M. P. Dobson M. Zhang C. Zhang M. Lion C. Gutiérrez-Martín C. B.. (2007). Genomic deletion marking an emerging subclone of *Francisella tularensis* subsp. holarctica in France and the Iberian Peninsula. Appl. Environ. Microbiol. 73, 7465–7470. 10.1128/AEM.00646-07 17890329 PMC2168206

[B25] Dennis D. T. Inglesby T. V. Henderson D. A. Bartlett J. G. Ascher M. S. Eitzen E.. (2001). Tularemia as a biological weapon: medical and public health management. JAMA 285, 2763–2773. 10.1001/jama.285.21.2763 11386933

[B26] Dentan C. Pavese P. Pelloux I. Boisset S. Brion J.-P. Stahl J.-P.. (2013). Treatment of tularemia in pregnant woman, France. Emerging Infect. Dis. 19, 996–998. 10.3201/eid1906.130138 23735285 PMC3713841

[B27] El-Etr S. H. Margolis J. J. Monack D. Robison R. A. Cohen M. Moore E.. (2009). *Francisella tularensis* type A strains cause the rapid encystment of *Acanthamoeba castellanii* and survive in amoebal cysts for three weeks postinfection. Appl. Environ. Microbiol. 75, 7488–7500. 10.1128/AEM.01829-09 19820161 PMC2786426

[B28] Enderlin G. Morales L. Jacobs R. F. Cross J. T. (1994). Streptomycin and alternative agents for the treatment of tularemia: review of the literature. Clin. Infect. Dis. 19, 42–47. 10.1093/clinids/19.1.42 7948556

[B29] Erdem H. Ozturk-Engin D. Yesilyurt M. Karabay O. Elaldi N. Celebi G.. (2014). Evaluation of tularaemia courses: a multicentre study from Turkey. Clin. Microbiol. Infect. 20, O1042–O1051. 10.1111/1469-0691.12741 24975504

[B30] Feldman K. A. Enscore R. E. Lathrop S. L. Matyas B. T. McGuill M. Schriefer M. E.. (2001). An outbreak of primary pneumonic tularemia on Martha's Vineyard. N. Engl. J. Med. 345, 1601–1606. 10.1056/NEJMoa011374 11757506

[B31] Francis E. (1928). A Summary of Present Knowledge of Tularaemia1. Medicine 7, 411–432.

[B32] Francis E. Mayne B. Lake G. C. (1922). Tularæmia Francis 1921 a New Disease of Man. Washington, DC: Government Printing office.

[B33] Fujita O. Uda A. Hotta A. Okutani A. Inoue S. Tanabayashi K.. (2008). Genetic diversity of *Francisella tularensis* subspecies holarctica strains isolated in Japan. Microbiol. Immunol. 52, 270–276. 10.1111/j.1348-0421.2008.00036.x 18557897

[B34] García del Blanco N. Gutiérrez Martín C. B. de la Puente Redondo V. A. Rodríguez Ferri E. F. (2004). *In vitro* susceptibility of field isolates of *Francisella tularensis* subsp. holarctica recovered in Spain to several antimicrobial agents. Res. Vet. Sci. 76, 195–198. 10.1016/j.rvsc.2003.12.002 15046952

[B35] Georgi E. Schacht E. Scholz H. C. Splettstoesser W. D. (2012). Standardized broth microdilution antimicrobial susceptibility testing of *Francisella tularensis* subsp. holarctica strains from Europe and rare Francisella species J. Antimicrob. Chemother. 67, 2429–2433. 10.1093/jac/dks238 22763567

[B36] Golovliov I. Baranov V. Krocova Z. Kovarova H. Sjöstedt A. (2003). An attenuated strain of the facultative intracellular bacterium *Francisella tularensis* can escape the phagosome of monocytic cells. Infect. Immun. 71, 5940–5950. 10.1128/IAI.71.10.5940-5950.2003 14500514 PMC201066

[B37] Gyuranecz M. Birdsell D. N. Splettstoesser W. Seibold E. Beckstrom-Sternberg S. M. Makrai L.. (2012). Phylogeography of *Francisella tularensis* subsp. holarctica, Europe. Emerg. Infect. Dis. 18, 290–293. 10.3201/eid1802.111305 22305204 PMC3310461

[B38] Gyuranecz M. Rigó K. Dán A. Földvári G. Makrai L. Dénes B.. (2011). Investigation of the ecology of *Francisella tularensis* during an inter-epizootic period. Vector Borne Zoonotic. Dis. 11, 1031–1035. 10.1089/vbz.2010.0091 21142970

[B39] Hall I. H. Schwab U. E. Stacy Ward E. Butts J. D. Wolford E. T. Ives T. J. (2002). Disposition and intracellular activity of azithromycin in human THP-1 acute monocytes. Int. J. Antimicrob. Agents 20, 348–360. 10.1016/S0924-8579(02)00187-5 12431870

[B40] Hand W. L. Hand D. L. (2001). Characteristics and mechanisms of azithromycin accumulation and efflux in human polymorphonuclear leukocytes. Int. J. Antimicrob. Agents 18, 419–425. 10.1016/S0924-8579(01)00430-7 11711255

[B41] Hauri A. M. Hofstetter I. Seibold E. Kaysser P. Eckert J. Neubauer H.. (2010). Investigating an airborne tularemia outbreak, Germany. Emerging Infect. Dis. 16, 238–243. 10.3201/eid1602.081727 20113553 PMC2957990

[B42] Helvaci S. Gedikoğlu S. Akalin H. Oral H. B. (2000). Tularemia in Bursa, Turkey: 205 cases in ten years. Eur. J. Epidemiol. 16, 271–276. 10.1023/A:1007610724801 10870943

[B43] Hofinger D. M. Cardona L. Mertz G. J. Davis L. E. (2009). TUlaremic meningitis in the united states. Arch. Neurol. 66, 523–527. 10.1001/archneurol.2009.14 19364939

[B44] Hotta A. Fujita O. Uda A. Sharma N. Tanabayashi K. Yamamoto Y.. (2013). *In vitro* antibiotic susceptibility of *Francisella tularensis* isolates from Japan. Jpn. J. Infect. Dis. 66, 534–536. 10.7883/yoken.66.534 24270145

[B45] Huber B. Escudero R. Busse H.-J. Seibold E. Scholz H. C. Anda P.. (2010). Description of *Francisella hispaniensis* sp. nov., isolated from human blood, reclassification of *Francisella novicida* (Larson et al. 1955) Olsufiev et al. 1959 as *Francisella tularensis* subsp. novicida comb. nov. and emended description of the genus Francisella. Int. J. Syst. Evol. Microbiol. 60, 1887–1896. 10.1099/ijs.0.015941-0 19783615

[B46] Ikäheimo I. Syrjälä H. Karhukorpi J. Schildt R. Koskela M. (2000). *In vitro* antibiotic susceptibility of *Francisella tularensis* isolated from humans and animals. J. Antimicrob. Chemother. 46, 287–290. 10.1093/jac/46.2.287 10933655

[B47] Jellison W. L. (1974). Tularemia in North America, 1930-1974. University of Montana, University of Montana Foundation.

[B48] Jellison W. L. Owen C. R. (1961). Tularemia and Animal Populations Ecology and Epizootiology. West Salem, Wis.

[B49] Johansson A. Berglund L. Gothefors L. Sjöstedt A. Tärnvik A. (2000). Ciprofloxacin for treatment of tularemia in children. Pediatr. Infect. Dis. J. 19, 449–453. 10.1097/00006454-200005000-00011 10819342

[B50] Johansson A. Celli J. Conlan W. Elkins K. L. Forsman M. Keim P. S.. (2010). Objections to the transfer of *Francisella novicida* to the subspecies rank of *Francisella tularensis*. Int. J. Syst. Evol. Microbiol. 60, 1717–1720. 10.1099/ijs.0.022830-0 20688748 PMC7442299

[B51] Johansson A. Lärkeryd A. Widerström M. Mörtberg S. Myrtännäs K. Ohrman C.. (2014). A respiratory tularemia outbreak caused by diverse clones of *Francisella tularensis*. Clin. Infect. Dis. 59, 1546–1553. 10.1093/cid/ciu621 25097081 PMC4650766

[B52] Johansson A. Urich S. K. Chu M. C. Sjöstedt A. Tärnvik A. (2002). *In vitro* susceptibility to quinolones of *Francisella tularensis* subspecies *tularensis*. Scand. J. Infect. Dis. 34, 327–330. 10.1080/00365540110080773 12069013

[B53] Kantardjiev T. Ivanov I. Velinov T. Padeshki P. Popov B. Nenova R.. (2006). Tularemia outbreak, Bulgaria, 1997-2005. Emerging Infect. Dis. 12, 678–680. 10.3201/eid1204.050709 16704820 PMC3294687

[B54] Karlsson E. Golovliov I. Lärkeryd A. Granberg M. Larsson E. Öhrman C.. (2016). Clonality of erythromycin resistance in *Francisella tularensis*. J. Antimicrob. Chemother. 71, 2815–2823. 10.1093/jac/dkw235 27334667

[B55] Karlsson E. Svensson K. Lindgren P. Byström M. Sjödin A. Forsman M.. (2013). The phylogeographic pattern of *Francisella tularensis* in Sweden indicates a Scandinavian origin of *Eurosiberian tularaemia*. Environ. Microbiol. 15, 634–645. 10.1111/1462-2920.12052 23253075

[B56] Keim P. Johansson A. Wagner D. M. (2007). Molecular epidemiology, evolution, and ecology of Francisella. Ann. N. Y. Acad. Sci. 1105, 30–66. 10.1196/annals.1409.011 17435120

[B57] Kilic S. Birdsell D. N. Karagöz A. Çelebi B. Bakkaloglu Z. Arikan M.. (2015). Water as source of *Francisella tularensis* infection in humans, Turkey. Emerg. Infect. Dis. 21, 2213–2216. 10.3201/eid2112.150634 26583383 PMC4672436

[B58] Kiliç S. Çelebi B. Acar B. Ataş M. (2013). *In vitro* susceptibility of isolates of *Francisella tularensis* from Turkey. Scand. J. Infect. Dis. 45, 337–341. 10.3109/00365548.2012.751125 23249114

[B59] Kingry L. C. Petersen J. M. (2014). Comparative review of *Francisella tularensis* and *Francisella novicida*. Front. Cell. Infect. Microbiol. 4:35. 10.3389/fcimb.2014.00035 24660164 PMC3952080

[B60] Klimpel G. R. Eaves-Pyles T. Moen S. T. Taormina J. Peterson J. W. Chopra A. K.. (2008). Levofloxacin rescues mice from lethal intra-nasal infections with virulent *Francisella tularensis* and induces immunity and production of protective antibody. Vaccine 26, 6874–6882. 10.1016/j.vaccine.2008.09.077 18930100 PMC2630585

[B61] Kreizinger Z. Makrai L. Helyes G. Magyar T. Erdélyi K. Gyuranecz M. (2013). Antimicrobial susceptibility of *Francisella tularensis* subsp. holarctica strains from hungary, Central Europe. J. Antimicrob. Chemother. 68, 370–373. 10.1093/jac/dks399 23065699

[B62] Kudelina R. I. Olsufiev N. G. (1980). Sensitivity to macrolide antibiotics and lincomycin in *Francisella tularensis* holarctica. J. Hyg. Epidemiol. Microbiol. Immunol. 24, 84–91. 7190590

[B63] Kugeler K. J. Mead P. S. Janusz A. M. Staples J. E. Kubota K. A. Chalcraft L. G.. (2009). Molecular epidemiology of *Francisella tularensis* in the United States. Clin. Infect. Dis. 48, 863–870. 10.1086/597261 19245342

[B64] Larssen K. W. Afset J. E. Heier B. T. Krogh T. Handeland K. Vikøren T.. (2011). Outbreak of tularaemia in central Norway, January to March 2011. Euro Surveill. 16:19828. Available online at: www.eurosurveillance.org/ViewArticle.aspx?ArticleId=19828 21489376

[B65] Larssen K. W. Bergh K. Heier B. T. Vold L. Afset J. E. (2014). All-time high tularaemia incidence in Norway in 2011: report from the national surveillance. Eur. J. Clin. Microbiol. Infect. Dis. 33, 1919–1926. 10.1007/s10096-014-2163-2 24874046

[B66] Livermore D. M. (2003). Linezolid *in vitro*: mechanism and antibacterial spectrum. J. Antimicrob. Chemother 51, ii9–ii16. 10.1093/jac/dkg249 12730138

[B67] Madrid P. B. Chopra S. Manger I. D. Gilfillan L. Keepers T. R. Shurtleff A. C.. (2013). A systematic screen of FDA-approved drugs for inhibitors of biological threat agents. PLoS ONE 8:e60579. 10.1371/journal.pone.0060579 23577127 PMC3618516

[B68] Mailles A. Madani N. Maurin M. Garin-Bastuji B. Vaillant V. (2010). Unexpected increase of human and animal tularemia cases during winter 2007/2008 in France: emergence or short-lasting episode?. Med. Mal. Infect. 40, 279–284. 10.1016/j.medmal.2009.11.006 20044227

[B69] Matzneller P. Krasniqi S. Kinzig M. Sörgel F. Hüttner S. Lackner E.. (2013). Blood, tissue, and intracellular concentrations of azithromycin during and after end of therapy. Antimicrob. Agents Chemother. 57, 1736–1742. 10.1128/AAC.02011-12 23357769 PMC3623349

[B70] Maurin M. Gyuranecz M. (2016). Tularaemia: clinical aspects in Europe. Lancet Infect. Dis. 16, 113–124. 10.1016/S1473-3099(15)00355-2 26738841

[B71] Maurin M. Mersali N. F. Raoult D. (2000). Bactericidal activities of antibiotics against intracellular *Francisella tularensis*. Antimicrob. Agents Chemother. 44, 3428–3431. 10.1128/AAC.44.12.3428-3431.2000 11083651 PMC90216

[B72] Maurin M. Pelloux I. Brion J. P. Banõ J.-N. D. Picard A. (2011). Human tularemia in France, 2006–2010. Clin. Infect. Dis. 53, e133–e141. 10.1093/cid/cir612 22002987

[B73] Maurin M. Raoult D. (2001). Use of aminoglycosides in treatment of infections due to intracellular bacteria. Antimicrob. Agents Chemother. 45, 2977–2986. 10.1128/AAC.45.11.2977-2986.2001 11600345 PMC90771

[B74] Mccoy G. W. Chapin C. W. (1912). Further observations on a plague-like disease of rodents with a preliminary note on the causative agent, bacterium tularense. J. Infect. Dis. 10, 61–72. 10.1093/infdis/10.1.61

[B75] McDonald P. J. Pruul H. (1991). Phagocyte uptake and transport of azithromycin. Eur. J. Clin. Microbiol. Infect. Dis. 10, 828–833. 10.1007/BF01975835 1662626

[B76] Mengeloglu Z. Duran A. Hakyemez I. N. Ocak T. Kücükbayrak A. Karadag M.. (2014). Evaluation of patients with Tularemia in bolu province in northwestern Anatolia, Turkey. J. Infect. Dev. Ctries. 8, 315–319. 10.3855/jidc.3952 24619262

[B77] Meric M. Willke A. Finke E.-J. Grunow R. Sayan M. Erdogan S.. (2008). Evaluation of clinical, laboratory, and therapeutic features of 145 tularemia cases: the role of quinolones in oropharyngeal tularemia. APMIS 116, 66–73. 10.1111/j.1600-0463.2008.00901.x 18254782

[B78] Muller W. Hotzel H. Otto P. Karger A. Bettin B. Bocklisch H.. (2013). German *Francisella tularensis* isolates from European brown hares (*Lepus europaeus*) reveal genetic and phenotypic diversity. BMC Microbiol. 13:61. 10.1186/1471-2180-13-61 23517149 PMC3663675

[B79] Nano F. E. Schmerk C. (2007). The francisella pathogenicity Island. Ann. N. Y. Acad. Sci. 1105, 122–137. 10.1196/annals.1409.000 17395722

[B80] Olsufiev N. G. Emelyanova O. S. Dunayeva T. N. (1959). Comparative study of strains of *B. tularense* in the old and new world and their taxonomy. J. Hyg. Epidemiol. Microbiol. Immunol. 3, 138–149. 14428832

[B81] Olsufjev N. G. (1970). Taxonomy and characteristic of the genus Francisella Dorofeev, 1947. J. Hyg. Epidemiol. Microbiol. Immunol. 14, 67–74. 5462048

[B82] Olsufjev N. G. Meshcheryakova I. S. (1982). Infraspecific taxonomy of tularemia agent *Francisella tularensis* McCoy et Chapin. J. Hyg. Epidemiol. Microbiol. Immunol. 26, 291–299. 7142690

[B83] Olsufjev N. G. Meshcheryakova I. S. (1983). Subspecific Taxonomy of *Francisella tularensis* McCoy and Chapin 1912. Int. J. Syst. Bacteriol. 33, 872–874. 10.1099/00207713-33-4-872

[B84] Origgi F. C. Frey J. Pilo P. (2014). Characterisation of a new group of *Francisella tularensis* subsp. holarctica in Switzerland with altered antimicrobial susceptibilities, 1996 to 2013. Euro Surveill. 19:20858. 10.2807/1560-7917.ES2014.19.29.20858 25080140

[B85] Payne L. Arneborn M. Tegnell A. Giesecke J. (2005). Endemic tularemia, Sweden, 2003. Emerging Infect. Dis. 11, 1440–1442. 10.3201/eid1109.041189 16229776 PMC3310613

[B86] Pérez-Castrillón J. L. Bachiller-Luque P. Martín-Luquero M. Mena-Martín F. J. Herreros V. (2001). Tularemia epidemic in Northwestern Spain: clinical description and therapeutic response. Clin. Infect. Dis. 33, 573–576. 10.1086/322601 11462198

[B87] Petersen J. M. Carlson J. K. Dietrich G. Eisen R. J. Coombs J. Janusz A. M.. (2008). Multiple *Francisella tularensis* subspecies and clades, tularemia outbreak, utah. Emerg. Infect. Dis. 14, 1928–1930. 10.3201/eid1412.080482 19046524 PMC2634622

[B88] Piercy T. Steward J. Lever M. S. Brooks T. J. G. (2005). *In vivo* efficacy of fluoroquinolones against systemic tularaemia infection in mice. J. Antimicrob. Chemother. 56, 1069–1073. 10.1093/jac/dki359 16223941

[B89] Pilo P. Johansson A. Frey J. (2009). Identification of *Francisella tularensis* cluster in central and Western Europe. Emerg. Infect. Dis. 15, 2049–2051. 10.3201/eid1512.080805 19961699 PMC3044507

[B90] Raymond C. R. Conlan J. W. (2009). Differential susceptibility of Sprague-Dawley and Fischer 344 rats to infection by *Francisella tularensis*. Microb. Pathog. 46, 231–234. 10.1016/j.micpath.2009.01.002 19490832

[B91] Reintjes R. Dedushaj I. Gjini A. Jorgensen T. R. Cotter B. Lieftucht A.. (2002). Tularemia outbreak investigation in Kosovo: case control and environmental studies. Emerg. Infect. Dis. 8, 69–73. 10.3201/eid0801.010131 11749751 PMC2730257

[B92] Rick Lyons C. Wu T. H. (2007). Animal models of *Francisella tularensis* infection. Ann. N. Y. Acad. Sci. 1105, 238–265. 10.1196/annals.1409.003 17395735

[B93] Rockwood S. (1983). Tularemia: what's in a name. ASM News 63–65.

[B94] Rotem S. Bar-Haim E. Cohen H. Elia U. Ber R. Shafferman A.. (2012). Consequences of delayed ciprofloxacin and doxycycline treatment regimens against *Francisella tularensis* airway infection. Antimicrob. Agents Chemother. 56, 5406–5408. 10.1128/AAC.01104-12 22850512 PMC3457351

[B95] Russell P. Eley S. M. Fulop M. J. Bell D. L. Titball R. W. (1998). The efficacy of ciprofloxacin and doxycycline against experimental tularaemia. J. Antimicrob. Chemother. 41, 461–465. 10.1093/jac/41.4.461 9598777

[B96] Scheel O. Hoel T. Sandvik T. Berdal B. P. (1993). Susceptibility pattern of scandinavian *Francisella tularensis* isolates with regard to oral and parenteral antimicrobial agents. APMIS 101, 33–36. 10.1111/j.1699-0463.1993.tb00077.x 8384458

[B97] Schmitt D. M. O'Dee D. M. Cowan B. N. Birch J. W.-M. Mazzella L. K. Nau G. J.. (2013). The use of resazurin as a novel antimicrobial agent against *Francisella tularensis*. Front. Cell. Infect. Microbiol. 3:93. 10.3389/fcimb.2013.00093 24367766 PMC3853850

[B98] Siret V. Barataud D. Prat M. Vaillant V. Ansart S. Le Coustumier A.. (2006). An outbreak of airborne tularaemia in France, August 2004. Euro Surveill. 11, 58–60. 16525197

[B99] Sjostedt A. (2007). Tularemia: history, epidemiology, pathogen physiology, and clinical manifestations. Ann. N. Y. Acad. Sci. 1105, 1–29. 10.1196/annals.1409.009 17395726

[B100] Stundick M. V. Albrecht M. T. Houchens C. R. Smith A. P. Dreier T. M. Larsen J. C. (2013). Animal models for *Francisella tularensis* and *Burkholderia* Species scientific and regulatory gaps toward approval of antibiotics under the FDA animal rule. Vet. Pathol. 50, 877–892. 10.1177/0300985813486812 23628693

[B101] Sutera V. Caspar Y. Boisset S. Maurin M. (2014). A new dye uptake assay to test the activity of antibiotics against intracellular *Francisella tularensis*. Front. Cell. Infect. Microbiol. 4:36. 10.3389/fcimb.2014.00036 24672776 PMC3957058

[B102] Svensson K. Back E. Eliasson H. Berglund L. Granberg M. Karlsson L.. (2009a). Landscape epidemiology of tularemia outbreaks in Sweden. Emerg. Infect. Dis. 15, 1937–1947. 10.3201/eid1512.090487 19961673 PMC3044527

[B103] Svensson K. Granberg M. Karlsson L. Neubauerova V. Forsman M. Johansson A. (2009b). A real-time PCR array for hierarchical identification of francisella isolates. PLoS ONE 4:e8360. 10.1371/journal.pone.0008360 20027310 PMC2793073

[B104] Tarnvik A. (2007). WHO Guidelines on Tularaemia. Geneva: WHO/CDS/EPR/2007.7.

[B105] Tärnvik A. Berglund L. (2003). Tularaemia. Eur. Respir. J. 21, 361–373. 10.1183/09031936.03.00088903 12608453

[B106] Tärnvik A. Chu M. C. (2007). New approaches to diagnosis and therapy of tularemia. Ann. N. Y. Acad. Sci. 1105, 378–404. 10.1196/annals.1409.017 17468229

[B107] Tomaso H. Al Dahouk S. Hofer E. Splettstoesser W. D. Treu T. M. Dierich M. P.. (2005). Antimicrobial susceptibilities of Austrian *Francisella tularensis* holarctica biovar II strains. Int. J. Antimicrob. Agents 26, 279–284. 10.1016/j.ijantimicag.2005.07.003 16143497

[B108] Urich S. K. Petersen J. M. (2008). *In vitro* susceptibility of isolates of *Francisella tularensis* types A and B from North America. Antimicrob. Agents Chemother. 52, 2276–2278. 10.1128/AAC.01584-07 18411318 PMC2415761

[B109] Valade E. Vaissaire J. Mérens A. Hernandez E. Gros C. Doujet C. L. (2008). Susceptibility of 71 French isolates of *Francisella tularensis* subsp. holarctica to eight antibiotics and accuracy of the Etest® method. J. Antimicrob. Chemother. 62, 208–210. 10.1093/jac/dkn146 18397924

[B110] Velinov K. Kantardjiev T. Nicolova M. Kuzmanov A. (2011). *In vitro* antimicrobial susceptibility of *Francisella tularensis* isolated in Bulgaria. Probl. Infect. Parasit. Dis. 39, 7–9.

[B111] Vogler A. J. Birdsell D. Price L. B. Bowers J. R. Beckstrom-Sternberg S. M. Auerbach R. K.. (2009). Phylogeography of *Francisella tularensis*: global expansion of a highly fit clone. J. Bacteriol. 191, 2474–2484. 10.1128/JB.01786-08 19251856 PMC2668398

[B112] Wang Y. Hai R. Zhang Z. Xia L. Cai H. Liang Y.. (2011). Genetic relationship between *Francisella tularensis* strains from China and from other countries. Biomed. Environ. Sci. 24, 310–314. 10.3967/0895-3988.2011.03.015 21784318

[B113] Wang Y. Peng Y. Hai R. Xia L. Li H. Zhang Z.. (2014). Diversity of *Francisella tularensis* Subsp. *holarctica* Lineages, China. Emerg. Infect. Dis. 20, 1191–1194. 10.3201/eid2007.130931 24963721 PMC4073844

[B114] Wherry W. B. Lamb B. H. (1914). Infection of man with bacterium tularense. J. Infect. Dis. 15, 331–340. 10.1093/infdis/15.2.331 15108712

[B115] Yeşilyurt M. Kılıç S. Çelebi B. Çelik M. Gül S. Erdoğan F.. (2011). Antimicrobial susceptibilities of *Francisella tularensis* subsp. holarctica strains isolated from humans in the Central Anatolia region of Turkey. J. Antimicrob. Chemother. 66, 2588–2592. 10.1093/jac/dkr338 21856791

