# Supplementary material for: Francisella tularensis Susceptibility to Antibiotics: A Comprehensive Review of the Data Obtained In vitro and in Animal Models
Source: Front Cell Infect Microbiol. 2017 Apr 11;7:122. doi: 10.3389/fcimb.2017.00122 (PMC5386985; doi:10.3389/fcimb.2017.00122)
Supplement: Supplementary file 1 [file Table1.DOC]

Supplementary Material

***Francisella tularensis* susceptibility to antibiotics: a comprehensive review of data obtained *in vitro* and in animal models.**

**Yvan Caspar,^1,2,3*^ Max Maurin ^1,2,3^**

*** Corresponding author:** Dr. Yvan Caspar **:** YCaspar@chu-grenoble.fr

# Supplementary Figures and Tables

|  |  |  |  |  | MIC (mg/L) | | | | | | | | | | | | | | | |
| --- | --- | --- | --- | --- | --- | --- | --- | --- | --- | --- | --- | --- | --- | --- | --- | --- | --- | --- | --- | --- |
| Reference | Year(s) | Origin | Nb of strains Type/biovar | **Culture**  **medium** | Gentamicin | | Streptomycin | | Doxycycline | | Ciprofloxacin | | Levofloxacin | | Tetracycline | | Erythromycin | | Chloramphenicol | |
|  |  |  |  |  | Range | MIC_90_ | Range | MIC_90_ | Range | MIC_90_ | Range | MIC_90_ | Range | MIC_90_ | Range | MIC_90_ | Range | MIC_90_ | Range | MIC_90_ |
| Broth microdilution | |  |  |  |  |  |  |  |  |  |  |  |  |  |  |  |  |  |  |  |
| Origgi et al | 1996–2013 | Switzerland | 19 B FTNF002 | Modified MHII | 1–4 | 4 | 2–4 | 4 |  |  | ≤0.06 | ≤0.06 |  |  | 2–8 | 8 | 1–8 | 4 | ≤2 | ≤2 |
|  |  |  | 5 B13 |  | 2 |  | 2–4 |  |  |  | ≤0.06 |  |  |  | 4 |  | >32 |  | ≤2 |  |
| García del Blanco et al | 1997–1999 | Spain | 42 B | Modified MHII | 1–8 | 8 | 4–32 | 32 | 2->32 | >32 | 0.06–0.25 | 0.25 | ≤0.25 | ≤0.25 | 4–64 | 64 | 2->4 | >4 | ≤8 | ≤8 |
| Agar dilution | |  |  |  |  |  |  |  |  |  |  |  |  |  |  |  |  |  |  |  |
| Scheel et al | UNK | Scandinavia | 20 B | Blood cystein agar | 2–8 |  | 2–16 |  | 0.25–2 |  | 0.031–0.125 |  |  |  |  |  | **Range: 0.5–8 (7 strains) or >32 (13 strains)** | | 1–8 |  |

**Table S1: Characteristics and MICs of strains from studies excluded from the analysis**
